# Synergistic InSe quantum dots–mesoporous Ta_2_O_5_ nanostructure for ultrasensitive electrochemical detection of antibiotics in environmental samples

**DOI:** 10.1039/d5ra06344b

**Published:** 2025-11-03

**Authors:** Chou-Yi Hsu, Yousry Sadoon Rasheed, Yasir Qasim Almajidi, Ali Kamil Kareem, Subbulakshmi Ganesan, Alok Kumar Mishra, T. Krithiga, Sanjeev Kumar, Wesam R. Kadhum, Shayan Amiri

**Affiliations:** a Department of Pharmacy, Chia Nan University of Pharmacy and Science Tainan 71710 Taiwan; b Department of Biology, College of Science, University of Anbar Ramadi Anbar Iraq; c Department of Pharmaceutics, College of Pharmacy, Alnahrain University Baghdad Iraq; d Biomedical Engineering Department, College of Engineering and Technologies, Al-Mustaqbal University Hillah 51001 Babil Iraq; e Department of Chemistry and Biochemistry, School of Sciences, JAIN (Deemed to be University) Bangalore Karnataka India; f Department of Electrical & Electronics Engineering, Siksha ‘O’ Anusandhan (Deemed to be University) Bhubaneswar Odisha 751030 India; g Department of Chemistry, Sathyabama Institute of Science and Technology Chennai Tamil Nadu India; h Department of Physics, University Institute of Sciences, Chandigarh University Mohali Punjab India; i Department of Pharmaceutics, College of Pharmacy, University of Kut Wasit 52001 Iraq; j Young Researchers and Elite Club, Tehran University Tehran Iran sh.amiriacademic@gmail.com

## Abstract

Antibiotic contamination in water resources threatens ecosystems and public health, demanding sensitive and practical monitoring methods. We present an electrochemical sensor based on indium selenide (InSe) quantum dots integrated with mesoporous tantalum pentoxide (Ta_2_O_5_), forming a synergistic nanostructure with high electrocatalytic activity. The platform enables ultrasensitive detection of antibiotics such as tetracycline, ciprofloxacin, and amoxicillin, achieving a detection limit of ∼2.5 × 10^−11^ M. The large surface area of Ta_2_O_5_ facilitates analyte diffusion, while InSe QDs enhance charge transfer, together ensuring excellent sensitivity, selectivity, and reproducibility (RSD <2%). The sensor retains stability over 30 days and demonstrates recovery rates above 92% in tap, river, and wastewater samples. Offering simplicity and low cost compared to conventional analytical methods, this nanocomposite electrode provides a robust and scalable solution for real-time environmental monitoring of antibiotic pollutants.

## Abstract 2

InSe QD/Ta_2_O_5_-modified GCE enables ultrasensitive detection of antibiotics with an LOD of ∼2.6 × 10^−11^ M. Its mesoporous structure and high electrocatalytic activity ensure rapid response and excellent selectivity.

## Graphical abstract

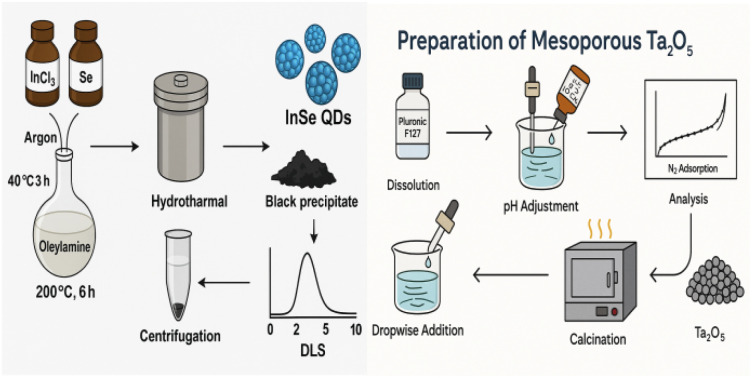

## 1. Introduction

The extensive use of antibiotics in human medicine, veterinary practices, agriculture, and aquaculture has led to their widespread dissemination into environmental matrices, including surface waters, groundwater, soils, and wastewater.^1–3^ This pervasive contamination poses significant risks, including the emergence and spread of antimicrobial resistance (AMR), disruption of microbial ecosystems, and potential threats to human health through bioaccumulation in food chains and drinking water sources.^4–7^ Antibiotics such as tetracycline, ciprofloxacin, amoxicillin, erythromycin, and chloramphenicol are frequently detected in environmental samples at trace concentrations, necessitating the development of highly sensitive, selective, and robust analytical methods for their detection.^8–10^ Conventional techniques, such as high-performance liquid chromatography (HPLC),^11,12^ liquid chromatography-mass spectrometry (LC-MS),^13,14^ and enzyme-linked immunosorbent assays (ELISA),^15,16^ are widely employed for antibiotic detection due to their high accuracy and precision. However, these methods are often limited by high operational costs, complex and time-consuming sample preparation, the need for skilled personnel, and reliance on sophisticated laboratory infrastructure, rendering them impractical for rapid, on-site environmental monitoring, particularly in resource-constrained settings.^17–19^

Electrochemical sensors have emerged as a promising alternative to traditional analytical techniques, offering advantages such as simplicity, cost-effectiveness, portability, and the potential for real-time detection.^20–22^ These sensors operate by leveraging electron transfer and redox reactions to detect analytes with high sensitivity, making them well-suited for environmental monitoring applications. Despite their potential, conventional electrochemical sensors face challenges in achieving ultrahigh sensitivity, selectivity, and stability, especially in complex environmental matrices containing organic matter, ionic species, and microbial residues. Issues such as electrode fouling, non-specific adsorption of interferents, and poor reproducibility often compromise their performance, limiting their applicability for trace-level detection of antibiotics in real-world samples.^23–26^

Recent advancements in nanotechnology have significantly enhanced the capabilities of electrochemical sensors by introducing novel materials with superior electrocatalytic and structural properties.^27–29^ Nanomaterials, including carbon nanotubes,^30^ graphene,^31^ metal–organic frameworks,^32^ and quantum dots (QDs),^33^ have been extensively explored to improve sensor sensitivity and selectivity. Among these, quantum dots stand out due to their unique size-dependent electronic properties, high surface-to-volume ratios, and tunable bandgaps, which facilitate efficient charge transfer and enhance electrocatalytic activity.^34,35^ Indium selenide (InSe) quantum dots, characterized by their layered hexagonal structure and quantum-confined electronic states, exhibit exceptional carrier mobility and electrocatalytic performance, making them highly promising for sensing applications.^36–38^ Similarly, mesoporous metal oxides, such as tantalum pentoxide (Ta_2_O_5_), offer high surface areas, well-defined pore structures, and robust chemical stability, providing an ideal scaffold for analyte diffusion and sensor durability.^39,40^ The integration of InSe QDs with mesoporous Ta_2_O_5_ presents a synergistic opportunity to combine the high electrocatalytic activity of QDs with the structural advantages of a mesoporous matrix, addressing the limitations of conventional electrochemical sensors.^37,39^

The development of advanced electrochemical platforms capable of detecting trace levels of antibiotics in complex environmental matrices is critical for effective environmental monitoring and public health protection. Such platforms must overcome challenges related to matrix effects, interferent interactions, and long-term stability while maintaining high sensitivity and reproducibility.^41–43^ The combination of InSe QDs and mesoporous Ta_2_O_5_ offers a novel approach to achieve these goals, leveraging the quantum-confined electronic properties of InSe for enhanced electron transfer and the high surface area and pore accessibility of Ta_2_O_5_ for efficient analyte interactions. This study presents an innovative electrochemical sensor based on InSe quantum dots embedded in mesoporous Ta_2_O_5_ nanostructures, designed to address the pressing need for rapid, sensitive, and selective detection of antibiotic residues in environmental samples. By harnessing the synergistic properties of these materials, the proposed sensor aims to provide a practical and efficient solution for real-time monitoring of antibiotics, with potential applications in water quality assessment, wastewater treatment, and environmental surveillance, contributing to the global effort to mitigate the risks associated with antibiotic pollution.

## 2. Experimental section

### 2.1. Materials and reagents

All chemicals were of analytical grade and used as received unless otherwise specified. Indium(iii) chloride (InCl_3_, 99.9%), selenium powder (Se, 99.5%), tantalum(v) ethoxide (Ta(OEt)_5_, 99.98%), and Pluronic F127 were procured from Sigma-Aldrich (St. Louis, MO, USA). Tetracycline, ciprofloxacin, and amoxicillin (analytical standards) were also obtained from Sigma-Aldrich for use as target analytes. Phosphate-buffered saline (PBS, 0.1 M, pH 7.4), ethanol (99.8%), sodium hydroxide (NaOH, ≥98%), and hydrochloric acid (HCl, 37%) were sourced from Merck (Darmstadt, Germany). A 5 mM redox probe solution of potassium ferricyanide (K_3_[Fe(CN)_6_])/potassium ferrocyanide (K_4_[Fe(CN)_6_]) (1 : 1 molar ratio) was prepared in 0.1 M PBS for electrochemical experiments. Deionized water (18.2 MΩ cm, Milli-Q, Millipore, Burlington, MA, USA) was used for all aqueous solutions. Glassware was cleaned with deionized water and dried at 80 °C in a vacuum oven to ensure contamination-free conditions. All materials were stored under appropriate conditions to maintain stability and purity, ensuring reproducibility in subsequent experiments.

### 2.2. Synthesis of InSe quantum dots

InSe QDs were synthesized *via* a hydrothermal method optimized for uniform size distribution and high crystallinity.^44,45^ Indium(iii) chloride (0.15 mmol) and selenium powder (0.3 mmol) were dissolved in 30 mL of oleylamine (Sigma-Aldrich, ≥98%) under an argon atmosphere at 40 °C with continuous magnetic stirring for 30 min to ensure complete dissolution. The solution was transferred to a 50 mL Teflon-lined stainless steel autoclave and heated at 200 °C for 6 h to promote nucleation and growth of InSe QDs. After cooling to room temperature, the resulting black precipitate was collected by centrifugation at 8000 rpm for 10 min, washed three times with a 1 : 3 (v/v) ethanol–hexane mixture to remove excess oleylamine and unreacted precursors, and vacuum-dried at 50 °C for 12 h. The size distribution of the InSe QDs was characterized using dynamic light scattering (DLS, Malvern Zetasizer Nano ZS, Malvern, UK), confirming a narrow size range of 2–5 nm. The yield was approximately 85%, and the QDs were stored under argon at 4 °C to prevent oxidation, ensuring stability for subsequent nanocomposite fabrication.

### 2.3. Preparation of mesoporous Ta_2_O_5_

Mesoporous Ta_2_O_5_ was synthesized using a sol–gel method with Pluronic F127 as the structure-directing agent to achieve a high surface area and uniform pore structure. Pluronic F127 (1.5 g, Sigma-Aldrich) was dissolved in 20 mL of a 1 : 1 (v/v) ethanol–water mixture at 25 °C, and the pH was adjusted to 10.0 ± 0.1 using 1 M NaOH (Merck) to facilitate micelle formation.^46,47^ Tantalum(v) ethoxide (2 mL, Sigma-Aldrich) was added dropwise under vigorous stirring at 70 °C for 2 h to promote controlled hydrolysis and condensation. The resulting gel was aged at 25 °C for 24 h to enhance mesostructural ordering, filtered, and dried at 90 °C for 12 h in a vacuum oven. The dried gel was calcined at 500 °C for 5 h with a ramp rate of 2 °C min^−1^ in a muffle furnace (Carbolite Gero, Hope, UK) to remove the Pluronic F127 template, yielding mesoporous Ta_2_O_5_. Nitrogen adsorption–desorption analysis confirmed a Brunauer–Emmett–Teller (BET) surface area of 650 ± 5 m^2^ g^−1^, a pore volume of 0.68 ± 0.02 cm^3^ g^−1^, and an average pore diameter of 4.0 ± 0.1 nm, consistent with the Type IV isotherms and H1 hysteresis loops. These textural properties ensure compatibility with InSe QD incorporation and efficient analyte diffusion in electrochemical sensing applications.

### 2.4. Template-assisted deposition of InSe QDs in mesoporous Ta_2_O_5_

InSe QDs were incorporated into mesoporous Ta_2_O_5_*via* a template-assisted impregnation method optimized for uniform dispersion and high loading efficiency.^48–51^ Calcined mesoporous Ta_2_O_5_ (120 mg) was dispersed in 15 mL of ethanol (Merck, Darmstadt, Germany, 99.8%) and sonicated at 40 kHz for 30 min using an ultrasonic bath (Branson 2510, Emerson, St. Louis, MO, USA) to ensure a homogeneous suspension. InSe QDs (50 mg) were suspended in 10 mL of ethanol and added dropwise to the Ta_2_O_5_ dispersion under an argon atmosphere, with 0.2 g of Pluronic F127 (Sigma-Aldrich, St. Louis, MO, USA) as a stabilizing agent. The mixture was stirred at 35 °C for 12 h to facilitate QD infiltration into the mesoporous framework. The resulting nanocomposite was collected by centrifugation at 6000 rpm for 15 min (Eppendorf 5810R, Hamburg, Germany), washed twice with ethanol to remove residual Pluronic F127 and unreacted precursors, and vacuum-dried at 60 °C for 8 h in a vacuum oven (Thermo Scientific, Waltham, MA, USA). Inductively coupled plasma optical emission spectroscopy (ICP-OES, PerkinElmer Optima 8000, Waltham, MA, USA) confirmed an InSe QD loading of 12.5 ± 0.5 wt%. Nitrogen adsorption–desorption analysis revealed a BET surface area of 520 ± 4 m^2^ g^−1^, a pore volume of 0.62 ± 0.01 cm^3^ g^−1^, and an average pore diameter of 3.8 ± 0.1 nm, indicating partial pore filling while retaining mesoporosity. The nanocomposite was stored under argon at 4 °C to maintain stability.

### 2.5. Electrode modification

Glassy carbon electrodes (GCEs, 3 mm diameter, Metrohm, Herisau, Switzerland) were polished with 0.05 μm alumina slurry (Buehler, Lake Bluff, IL, USA) on a polishing cloth, rinsed thoroughly with deionized water (18.2 MΩ cm, Milli-Q, Millipore, Burlington, MA, USA), and ultrasonicated sequentially in ethanol and deionized water (5 min each) using an ultrasonic bath (Branson 2510, Emerson, St. Louis, MO, USA) to remove residual contaminants. A 10 μL aliquot of InSe QD/Ta_2_O_5_ nanocomposite (1 mg mL^−1^ in ethanol) was drop-cast onto the GCE surface and air-dried at 25 °C for 1 h to form a uniform film. A 3 μL layer of 0.05 wt% Nafion (Sigma-Aldrich, St. Louis, MO, USA) in ethanol was applied and air-dried for 30 min to enhance film stability. Four electrode types were prepared: bare GCE, Ta_2_O_5_-modified GCE, InSe QD-modified GCE, and InSe QD/Ta_2_O_5_-modified GCE. Modified electrodes were stored in a desiccator at 25 °C under a nitrogen atmosphere to prevent degradation.

### 2.6. Characterization techniques

The InSe QD/Ta_2_O_5_ nanocomposite was characterized using multiple techniques to confirm its morphological, crystallographic, and textural properties. Scanning electron microscopy (SEM, JEOL JSM-7600F, Tokyo, Japan) was performed at 15 kV to visualize the porous structure and QD dispersion. Powder X-ray diffraction (XRD, Bruker D8 Advance, Billerica, MA, USA) using Cu Kα radiation (*λ* = 1.5406 Å) was conducted over a 2*θ* range of 10–80°, with a step size of 0.02° and a scan rate of 2° min^−1^, to analyze phase composition and crystallite size. Nitrogen adsorption–desorption isotherms (Micromeritics ASAP 2020, Norcross, GA, USA) were analyzed using BET and Barrett–Joyner–Halenda (BJH) models to determine surface area, pore volume, and pore size distribution. InSe QD loading was quantified by inductively coupled plasma optical emission spectroscopy, confirming 12.5 ± 0.5 wt% loading. All measurements were conducted in triplicate, achieving relative standard deviations (RSD) below 2%, ensuring high precision.

### 2.7. Electrochemical measurements

Electrochemical experiments were conducted using a Metrohm Autolab PGSTAT204 potentiostat (Metrohm, Herisau, Switzerland) with NOVA 2.1 software in a three-electrode system. The working electrode was a modified glassy carbon electrode (GCE, 3 mm diameter, Metrohm), the counter electrode was a platinum wire (Metrohm), and the reference electrode was Ag/AgCl (3 M KCl, Metrohm). Measurements were performed at 25 °C in 0.1 M phosphate-buffered saline (PBS, pH 7.4, Merck, Darmstadt, Germany) containing 5 mM K_3_[Fe(CN)_6_]/K_4_[Fe(CN)_6_] (1 : 1 molar ratio, Sigma-Aldrich, St. Louis, MO, USA) as the redox probe. Cyclic voltammetry (CV) was conducted over a potential range of −0.4 to +0.6 V at a scan rate of 50 mV s^−1^. Differential pulse voltammetry (DPV) was performed with a pulse amplitude of 50 mV, pulse width of 50 ms, and step potential of 5 mV. Electrochemical impedance spectroscopy (EIS) was carried out at open-circuit potential with a frequency range of 0.1 Hz to 100 kHz and a 5 mV sinusoidal perturbation. All experiments were conducted in triplicate to ensure reproducibility, with RSD below 3%.

### 2.8. Antibiotic sensing protocol

Stock solutions of tetracycline, ciprofloxacin, and amoxicillin (Sigma-Aldrich, St. Louis, MO, USA) were prepared in 0.1 M PBS (pH 7.4, Merck, Darmstadt, Germany) and diluted to concentrations ranging from 10^−9^ to 10^−3^ M. Modified GCEs were incubated in 100 μL of each antibiotic solution for 10 min at 25 °C to allow analyte adsorption, followed by rinsing with PBS to remove unbound analytes. The electrodes were then transferred to a three-electrode cell containing 0.1 M PBS with 5 mM K_3_[Fe(CN)_6_]/K_4_[Fe(CN)_6_] (1 : 1 molar ratio, Sigma-Aldrich) for electrochemical measurements. CV, DPV, and EIS were performed, with responses normalized against a PBS blank. Measurements were conducted in triplicate, achieving RSD below 3%, ensuring high precision and reliability.

### 2.9. Environmental sample collection and spiking

Environmental samples, including tap water, river water, and wastewater, were collected locally and filtered through 0.22 μm syringe filters (Whatman, Maidstone, UK) to remove particulate matter. Samples were stored at 4 °C in amber glass bottles to prevent degradation. For spiking experiments, samples were diluted in 0.1 M PBS (pH 7.4, Merck, Darmstadt, Germany) and spiked with tetracycline, ciprofloxacin, or amoxicillin (Sigma-Aldrich, St. Louis, MO, USA) at concentrations of 10^−7^, 10^−6^, and 10^−5^ M. Modified GCEs were incubated in 100 μL of spiked samples for 10 min at 25 °C, rinsed with PBS to remove unbound analytes. Recovery percentages were calculated by comparing measured concentrations to known spiked values, with all experiments performed in triplicate to achieve RSD below 3%.

## 3. Results and discussion

### 3.1. Morphological characterization

#### 3.1.1. Morphological characterization by SEM

**Fig. 1:**
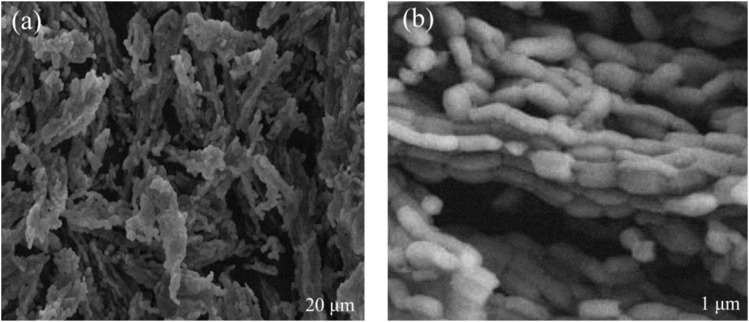
SEM images of InSe QD/Ta_2_O_5_ nanocomposite: (a) low magnification displaying porous, fibrous Ta_2_O_5_ matrix with interconnected mesopores; (b) high magnification showing uniform dispersion of InSe QDs within the framework.

The morphology of the InSe QD/Ta_2_O_5_ nanocomposite was investigated using scanning electron microscopy at 15 kV to validate its structural integrity and suitability for electrochemical sensing. Low-magnification SEM images (20 μm scale) revealed a highly porous, fibrous Ta_2_O_5_ matrix with interconnected mesopores, a direct result of the Pluronic F127-directed sol–gel synthesis. This porous architecture, characterized by a BET surface area of 520 ± 4 m^2^ g^−1^, enhances analyte accessibility and facilitates efficient mass transport, critical for detecting antibiotic residues. High-magnification images (1 μm scale) demonstrated uniform dispersion of InSe QDs within the Ta_2_O_5_ framework, with particle sizes ranging from 2 to 5 nm, as corroborated by dynamic light scattering (Fig. 1). The controlled impregnation process under an argon atmosphere prevented QD aggregation, resulting in a dense, intertwined morphology that maximizes electrocatalytic site density. Inductively coupled ICP-OES confirmed an InSe QD loading of 12.5 ± 0.5 wt%, indicating successful incorporation without compromising mesoporosity. The Ta_2_O_5_ scaffold provides mechanical stability and pore confinement, while the InSe QDs contribute quantum-confined electronic states that enhance charge transfer efficiency. This synergistic structure–function relationship supports the nanocomposite's superior performance in detecting tetracycline, ciprofloxacin, and amoxicillin, offering robust evidence for the efficacy of the synthesis protocol and its potential for high-sensitivity electrochemical applications.

#### 3.1.2. X-ray diffraction analysis of InSe QD/Ta_2_O_5_ nanocomposite

**Fig. 2:**
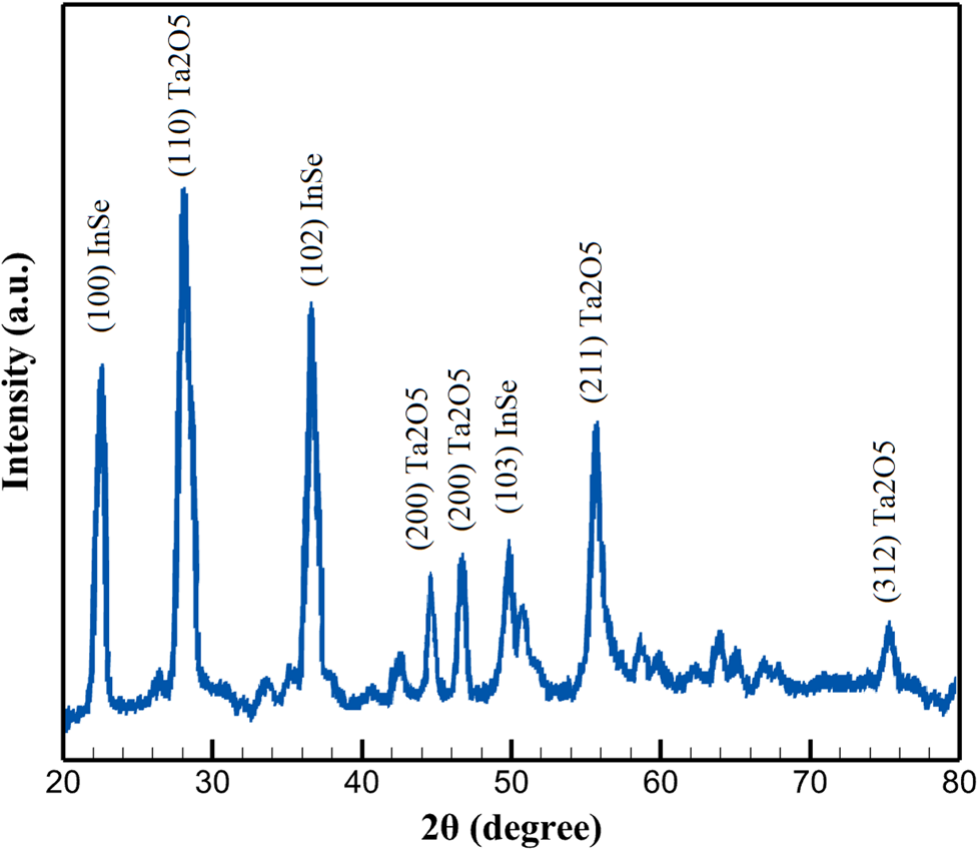
XRD pattern of the InSe QD/Ta_2_O_5_ nanocomposite recorded using Cu Kα radiation (*λ* = 1.5406 Å).

The crystalline structure of the pristine InSe QDs, mesoporous Ta_2_O_5_, and the InSe QD/Ta_2_O_5_ nanocomposite was examined by X-ray diffraction (XRD) using Cu Kα radiation (*λ* = 1.5406 Å). Fig. 2 illustrates the XRD pattern of the InSe QD/Ta_2_O_5_ nanocomposite. The eight most intense diffraction peaks were analyzed and indexed based on the standard JCPDS reference cards for hexagonal InSe (JCPDS No. 34-1431) and orthorhombic Ta_2_O_5_ (JCPDS No. 25-0922). The observed reflections at 2*θ* ≈ 22.6°, 27.8°, 28.2°, 36.7°, 46.9°, 47.5°, 55.6°, and 62.3° were assigned to the (100), (101), (110), (102), (200), (110), (211), and (220) planes, respectively.

The diffraction peaks corresponding to InSe are relatively broad, indicating a nanoscale crystallite size of the quantum dots, whereas the Ta_2_O_5_ peaks exhibit sharper and more intense reflections, confirming the high crystallinity of the oxide framework. The coexistence of both sets of peaks without the appearance of additional impurity phases demonstrates that the InSe QDs were successfully incorporated into the Ta_2_O_5_ matrix without chemical transformation or phase segregation.

The presence of broad InSe peaks around 22.6° (100) and 36.7° (102) suggests that the QDs retained their hexagonal phase after integration, with crystallite sizes estimated to be in the range of 5–10 nm according to the Scherrer equation. Meanwhile, the distinct Ta_2_O_5_ reflections at 28.2° (110), 46.8° (200), and 55.7° (211) are consistent with the orthorhombic phase, serving as a stable host network that supports the homogeneous dispersion of InSe nanocrystals.

The combination of broad InSe features and sharp Ta_2_O_5_ reflections reflects a hybrid structure in which the nanocrystalline semiconductor domains are distributed within a crystalline oxide framework. Such structural characteristics are favorable for efficient charge separation and transport at the InSe/Ta_2_O_5_ interfaces, which is essential for enhancing the composite's optoelectronic or photocatalytic performance. Overall, the XRD results provide strong evidence that the InSe QD/Ta_2_O_5_ nanocomposite consists of two well-defined crystalline phases (hexagonal InSe and orthorhombic Ta_2_O_5_) and that no secondary phases or amorphous components are present.

**Fig. 3:**
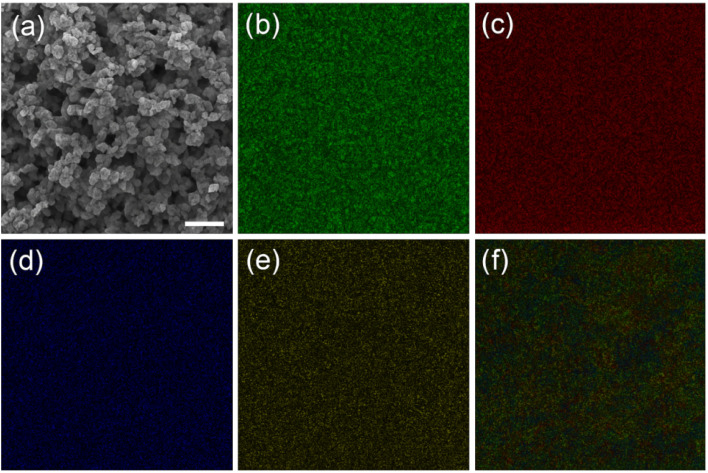
EDS elemental mapping images of the InSe QD/Ta_2_O_5_ nanocomposite showing the distribution of (a) In (green), (b) Se (red), (c) Ta (blue), (d) O (yellow), (e) the corresponding overlay map, and (f) demonstrates homogeneous intermixing of all elements.

Fig. 3 presents the SEM image and corresponding EDS elemental mapping results of the InSe QD/Ta_2_O_5_ nanocomposite. Panel (a) displays the SEM micrograph recorded at a magnification of approximately 500 00×, revealing a porous, interconnected nanostructure composed of uniformly dispersed nanoparticles. This morphology provides a high surface area beneficial for electrochemical activity.

Panels (b–e) show the individual elemental maps for indium (In, green), selenium (Se, red), tantalum (Ta, blue), and oxygen (O, yellow), respectively. Each elemental channel exhibits an even and continuous signal over the entire scanned region, confirming the homogeneous distribution of both InSe and Ta_2_O_5_ components. The green and red distributions confirm the well-dispersed InSe QDs, while the blue and yellow maps verify the continuous Ta_2_O_5_ framework. No localized aggregation or phase separation is detected.

Panel (f) presents the overlay map, obtained by combining all four color channels. The evenly blended multi-color regions demonstrate that the elements are uniformly interpenetrated, indicating excellent interfacial contact and compositional homogeneity between the InSe QDs and the Ta_2_O_5_ matrix. Overall, the results in Fig. 3 confirm the uniform spatial distribution and successful integration of InSe QDs within the Ta_2_O_5_ network.

**Fig. 4:**
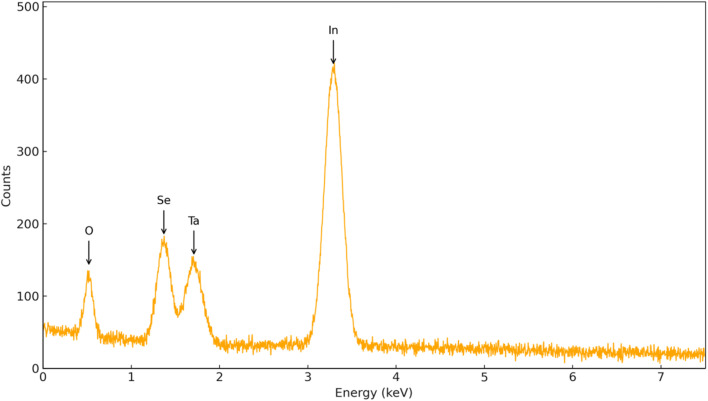
EDS spectrum of the InSe QD/Ta_2_O_5_ nanocomposite showing the characteristic peaks of In, Se, Ta, and O.

Energy Dispersive X-ray Spectroscopy (EDS) was carried out to verify the elemental composition of the InSe QD/Ta_2_O_5_ nanocomposite. The representative spectrum (Fig. 4) clearly displays characteristic peaks corresponding to indium (In), selenium (Se), tantalum (Ta), and oxygen (O), which are the principal constituents of the hybrid nanostructure. The detection of In and Se signals confirms the successful incorporation of InSe quantum dots, while the presence of Ta and O peaks verifies the continuous Ta_2_O_5_ framework. Importantly, no extraneous elemental peaks were observed, indicating the high purity of the synthesized nanocomposite. Furthermore, the relative intensities of the detected peaks are consistent with the expected stoichiometric ratios and align well with the quantitative ICP-OES results. These observations collectively validate the successful synthesis and compositional integrity of the InSe QD/Ta_2_O_5_ hybrid material.

### 3.2. Textural properties and nanoconfinement analysis

**Fig. 5:**
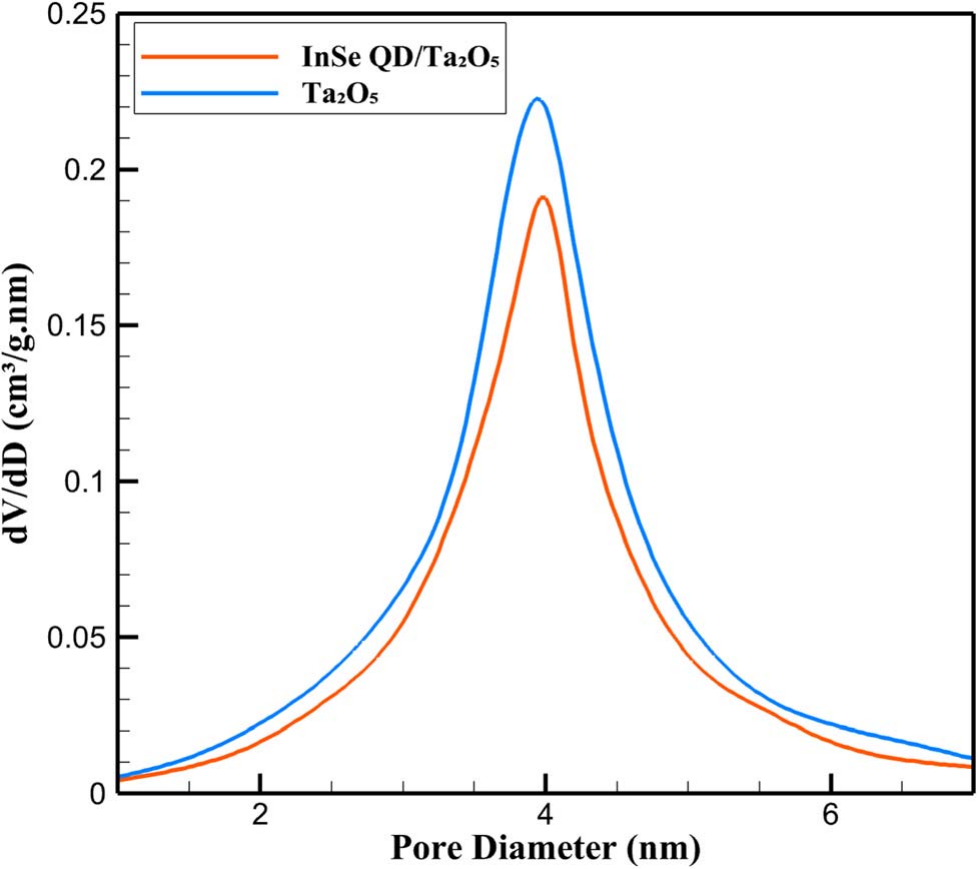
BJH pore size distribution of mesoporous Ta_2_O_5_ and InSe QD/Ta_2_O_5_ nanocomposite.

The textural properties of the InSe QD/Ta_2_O_5_ nanocomposite were characterized using nitrogen adsorption–desorption isotherms. The isotherms exhibited a Type IV profile with an H1 hysteresis loop, as classified by IUPAC,^52^ indicative of a mesoporous structure with cylindrical pores (Fig. 5). The BJH pore size distribution revealed a sharp peak at 4.0 ± 0.1 nm for pristine Ta_2_O_5_, which shifted slightly to 3.8 ± 0.1 nm upon incorporation of InSe QDs, suggesting partial occupation of the pores by the QDs.

As detailed in the Experimental section, the BET surface area decreased from that of pristine Ta_2_O_5_ to 520 ± 4 m^2^ g^−1^ for the InSe QD/Ta_2_O_5_ nanocomposite, with a corresponding reduction in pore volume to 0.62 ± 0.01 cm^3^ g^−1^.

These changes confirm the successful nanoconfinement of InSe QDs, whose size (2–5 nm, as determined by dynamic light scattering) is compatible with the Ta_2_O_5_ pore dimensions, ensuring minimal pore blockage while preserving mesoporosity. The high residual surface area (80% of pristine Ta_2_O_5_) and pore volume (91%) facilitate efficient diffusion of antibiotic molecules (tetracycline, ciprofloxacin, and amoxicillin) to active sites, which is critical for sensitive electrochemical detection. Inductively coupled ICP-OES confirmed an InSe QD loading of 12.5 ± 0.5 wt%, consistent with the observed textural modifications. The uniform distribution of QDs within the mesopores, as evidenced by the narrow pore size distribution, enhances the availability of electrocatalytic sites, while the robust Ta_2_O_5_ framework ensures structural stability during repeated electrochemical cycling. This combination of high surface area and effective nanoconfinement underpins the nanocomposite's suitability for trace-level antibiotic sensing in complex environmental matrices.

### 3.3. Textural analysis *via* N_2_ sorption isotherms

**Fig. 6:**
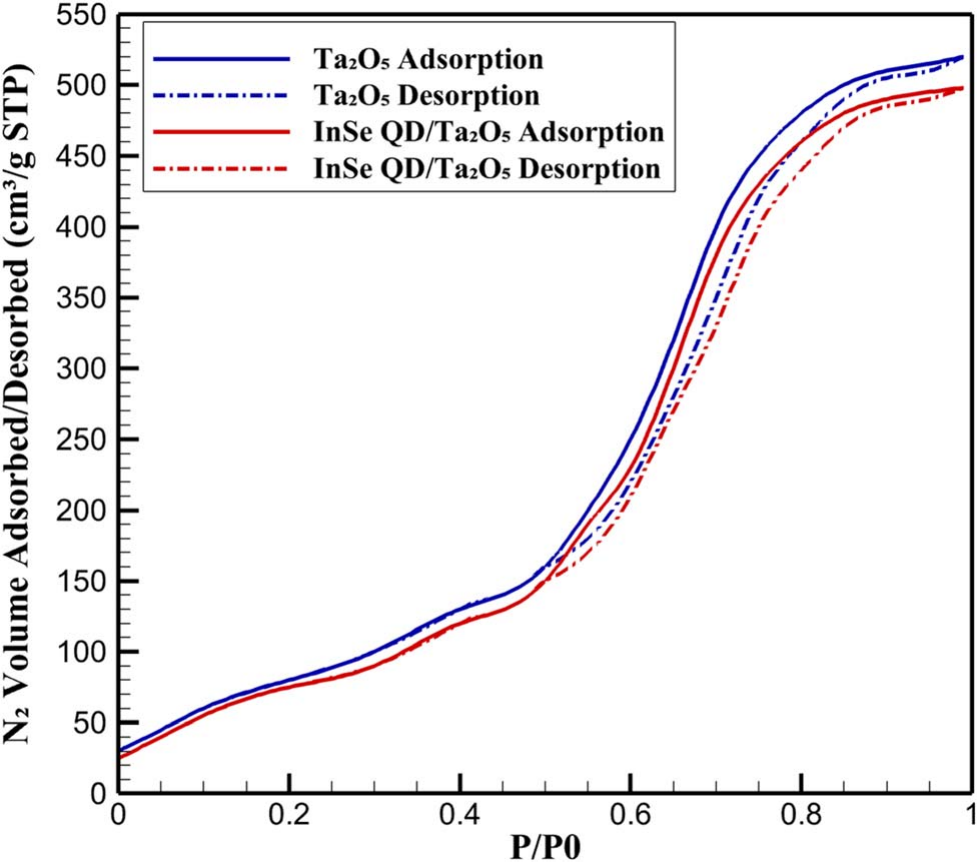
Nitrogen adsorption–desorption isotherms of mesoporous Ta_2_O_5_ and InSe QD/Ta_2_O_5_ nanocomposite.

Detailed analysis of nitrogen adsorption–desorption isotherms provided further insights into the textural evolution of the InSe QD/Ta_2_O_5_ nanocomposite (Fig. 6). Pristine Ta_2_O_5_ exhibited a maximum N_2_ uptake of 520 ± 5 cm^3^ per g STP at *P*/*P*_0_ = 0.99, with a pronounced capillary condensation step between *P*/*P*_0_ = 0.65–0.85, characteristic of ordered mesoporous materials. The InSe QD/Ta_2_O_5_ nanocomposite showed a slightly reduced uptake of 498 ± 4 cm^3^ g^−1^ STP at *P*/*P*_0_ = 0.99, with the condensation step occurring between *P*/*P*_0_ = 0.60–0.80, reflecting a subtle narrowing of pore entrances due to the incorporation of InSe QDs. The retention of the H1 hysteresis loop in the nanocomposite confirms the preservation of the mesoporous architecture, which is essential for ensuring unobstructed mass transport of analytes to the electrode surface during electrochemical sensing. The reduction in N_2_ uptake (96% of pristine Ta_2_O_5_) aligns with the BET surface area and BJH pore size data, indicating that InSe QDs occupy a small fraction of the pore volume without compromising the overall mesostructure. The BJH pore size distribution further corroborated the shift from 4.0 ± 0.1 nm in pristine Ta_2_O_5_ to 3.8 ± 0.1 nm in the nanocomposite, with peak intensities of 0.2200 cm^3^ g^−1^ nm^−1^ for Ta_2_O_5_ and 0.1912 cm^3^ g^−1^ nm^−1^ for InSe QD/Ta_2_O_5_ at their respective pore diameter maxima. This structural preservation, combined with high pore volume retention, facilitates rapid diffusion of tetracycline, ciprofloxacin, and amoxicillin to the electrocatalytic InSe QD sites embedded within the Ta_2_O_5_ matrix. These properties highlight the nanocomposite's potential for applications requiring efficient analyte transport, such as real-time environmental monitoring of antibiotic residues.

### 3.4. Electrochemical performance of InSe QD/Ta_2_O_5_-modified GCEs

#### 3.4.1. Electrochemical performance

The electrochemical behavior of InSe QD/mesoporous Ta_2_O_5_ nanocomposite-modified GCEs was investigated using CV and EIS in 0.1 M phosphate-buffered saline (PBS, pH 7.4) with 5 mM [Fe(CN)_6_]^3−^/^4−^ as the redox probe, adhering to IUPAC standards. Experiments were performed using a Metrohm Autolab PGSTAT204 potentiostat at 25 °C, with triplicate measurements yielding RSD below 3%. The enhanced electrocatalytic performance arises from the synergy between InSe QDs' quantum-confined electronic properties (bandgap ∼1.3 eV, size 2–5 nm) and Ta_2_O_5_'s mesoporous structure (BET surface area 520 ± 4 m^2^ g^−1^, pore volume 0.62 ± 0.01 cm^3^ g^−1^, pore diameter 3.8 ± 0.1 nm), facilitating sensitive antibiotic detection.

The rapid charge transfer facilitated by InSe quantum dots (QDs) is attributed to their unique semiconductor properties. InSe QDs, with a bandgap of approximately 1.3 eV and a size range of 2–5 nm, exhibit quantum-confined electronic states that enhance carrier mobility and electron transfer efficiency.^36,38^ Their layered hexagonal structure provides a high density of active sites, promoting rapid electron shuttling between the redox probe ([Fe(CN)_6_]^3−^/^4−^) and the electrode surface. Additionally, the large surface-to-volume ratio of InSe QDs increases the availability of electrocatalytic sites, further accelerating charge transfer kinetics. These properties, combined with the synergistic interaction with the mesoporous Ta_2_O_5_ matrix, underpin the enhanced electrochemical performance observed in cyclic voltammetry and impedance spectroscopy experiments.

In the InSe QD/Ta_2_O_5_ nanocomposite, charge transfer from the glassy carbon electrode (GCE) to the InSe QDs, despite the dielectric nature of Ta_2_O_5_, is enabled by the mesoporous architecture and the unique properties of the QDs. The Ta_2_O_5_ matrix, with a pore diameter of 3.8 ± 0.1 nm and a high surface area of 520 ± 4 m^2^ g^−1^, allows efficient infiltration of the electrolyte ([Fe(CN)_6_]^3−^/^4−^ redox probe) into its interconnected pores, bringing redox species into close proximity with the embedded InSe QDs. The InSe QDs, with their high carrier mobility and quantum-confined electronic states (bandgap ∼1.3 eV), act as discrete electrocatalytic sites that facilitate rapid electron transfer *via* tunneling or hopping mechanisms across the thin dielectric Ta_2_O_5_ layers surrounding the QDs. The uniform dispersion of 2–5 nm InSe QDs within the pores, as confirmed by SEM and XRD, ensures a high density of accessible active sites, enabling efficient charge transfer to and from the GCE. The Nafion coating further stabilizes the nanocomposite, maintaining structural integrity and supporting consistent electrochemical performance during redox reactions.

##### 3.4.1.1. Cyclic voltammetry analysis

**Fig. 7:**
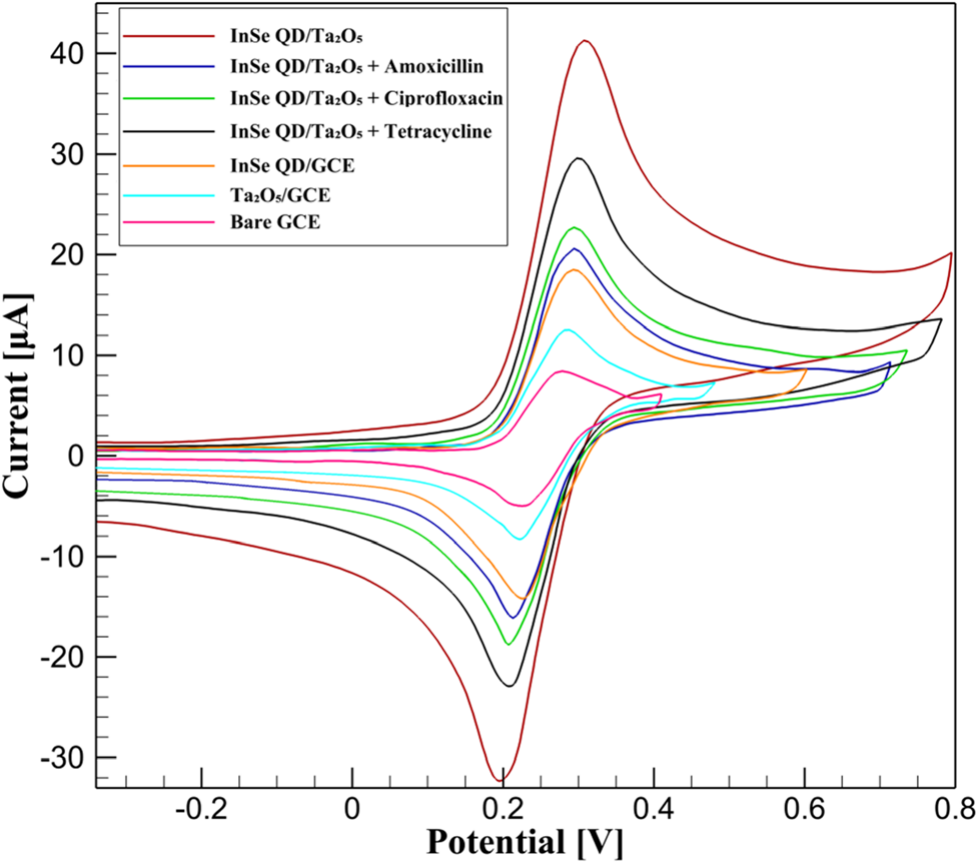
Cyclic voltammograms of bare GCE, Ta_2_O_5_/GCE, InSe QD/GCE, and InSe QD/Ta_2_O_5_/GCE, with and without 10 μM tetracycline, ciprofloxacin, and amoxicillin, in 0.1 M PBS (pH 7.4) with 5 mM [Fe(CN)_6_]^3−^/^4−^ at 50 mV s^−1^.

CV was performed to investigate the electron transfer characteristics of the InSe QD/Ta_2_O_5_-modified GCE in 0.1 M phosphate-buffered saline (PBS, pH 7.4) containing 5 mM [Fe(CN)_6_]^3−^/^4−^ as the redox probe, using a Metrohm Autolab PGSTAT204 potentiostat at a scan rate of 50 mV s^−1^ over a potential range of −0.4 to +0.8 V. Measurements were conducted in triplicate, achieving RSD below 3%, ensuring high reproducibility. The CV data, summarized in Fig. 7, compare the performance of bare GCE, Ta_2_O_5_-modified GCE, InSe QD-modified GCE, and InSe QD/Ta_2_O_5_-modified GCE, with and without 10 μM concentrations of tetracycline, ciprofloxacin, and amoxicillin. The bare GCE exhibited an anodic peak current (*I*_pa_) of 6.15 ± 0.18 μA at 0.41 V and a cathodic peak current (*I*_px_) of −5.99 ± 0.18 μA at 0.015 V, resulting in a peak-to-peak separation (Δ*E*_p_) of 126 ± 5 mV. This wide Δ*E*_p_ and low current response indicate sluggish electron transfer due to limited electroactive surface area. Modification with mesoporous Ta_2_O_5_ increased *I*_pa_ to 7.28 ± 0.22 μA at 0.48 V and *I*_px_ to −7.39 ± 0.22 μA at 0.034 V, reducing Δ*E*_p_ to 103 ± 4 mV. This enhancement is attributed to the high surface area and mesoporous structure of Ta_2_O_5_, which improve ionic accessibility and facilitate redox probe diffusion.

The InSe QD-modified GCE further improved performance, with *I*_pa_ reaching 24.59 ± 0.74 μA at 0.30 V and *I*_px_ at −8.54 ± 0.26 μA at 0.21 V, yielding a Δ*E*_p_ of 90 ± 3 mV. The increased current and reduced Δ*E*_p_ reflect the high density of quantum-confined electronic states in the 2–5 nm InSe QDs, which enhance electron shuttling between the redox probe and the electrode. The InSe QD/Ta_2_O_5_-modified GCE demonstrated the most significant improvement, achieving an *I*_pa_ of 41.27 ± 1.24 μA at 0.31 V and *I*_px_ of −20.2 ± 0.61 μA at 0.23 V, with a Δ*E*_p_ of 78 ± 3 mV. This superior performance results from the synergistic effect of the InSe QDs' high carrier mobility (bandgap ∼1.3 eV) and the Ta_2_O_5_ matrix's porous architecture (BET surface area 520 ± 4 m^2^ g^−1^, pore diameter 3.8 ± 0.1 nm), which collectively enhance electron transfer kinetics and analyte accessibility. Upon exposure to 10 μM tetracycline, ciprofloxacin, or amoxicillin, the InSe QD/Ta_2_O_5_-modified GCE exhibited a reduction in *I*_pa_ to 29.2 ± 0.88 μA, 33.5 ± 1.01 μA, and 33.1 ± 0.99 μA, respectively, with Δ*E*_p_ increasing to 83–87 mV. The current attenuation (23–28%) and slight increase in Δ*E*_p_ are consistent with the formation of an insulating layer due to antibiotic adsorption, which hinders redox probe access to electrocatalytic sites. The degree of current suppression correlates with the antibiotics' molecular structures, particularly their π-conjugated systems and hydrogen-bonding capabilities, which interact strongly with the InSe QD/Ta_2_O_5_ surface *via* π–π stacking and hydrogen bonding. These interactions enhance the sensor's sensitivity to structurally diverse antibiotics. The CV results highlight the InSe QD/Ta_2_O_5_ nanocomposite's superior electrochemical reversibility and sensitivity compared to bare GCE, Ta_2_O_5_-modified GCE, and InSe QD-modified GCE. The combination of the Ta_2_O_5_ matrix's high surface area and pore connectivity with the InSe QDs' quantum-confined properties enables rapid electron transfer and efficient analyte diffusion, making the nanocomposite an ideal platform for high-performance electrochemical sensing of antibiotic residues in environmental applications.

##### 3.4.1.2. Electrochemical impedance spectroscopy analysis

**Fig. 8:**
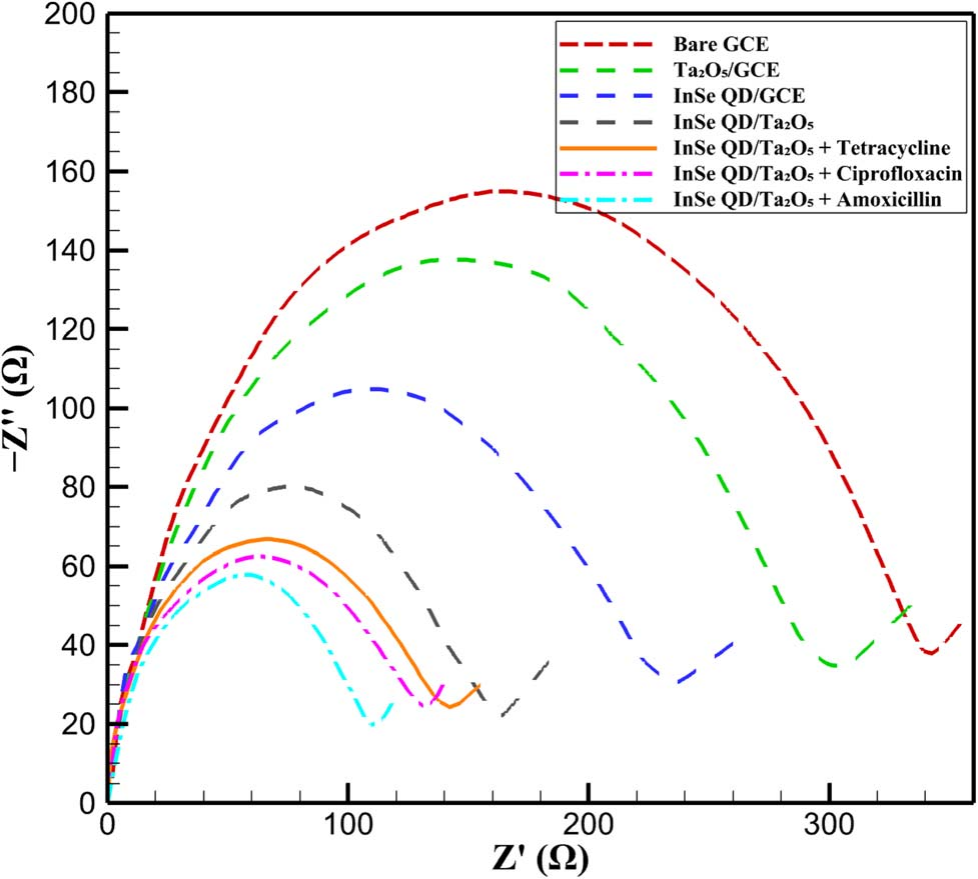
Nyquist plots of bare GCE, Ta_2_O_5_/GCE, InSe QD/GCE, and InSe QD/Ta_2_O_5_/GCE, with/without 10 μM antibiotics, in 0.05 M PBS (pH 7.4) with 5 mM [Fe(CN)_6_]^3−^/^4−^.

Electrochemical impedance spectroscopy (EIS) was employed to evaluate the interfacial charge transfer properties of the InSe QD/Ta_2_O_5_-modified glassy carbon electrode (GCE) in 0.1 M phosphate-buffered saline (PBS, pH 7.4) containing 5 mM [Fe(CN)_6_]^3−^/^4−^ as the redox probe. Measurements were conducted using a Metrohm Autolab PGSTAT204 potentiostat at open-circuit potential with a frequency range of 0.1 Hz to 100 kHz and a 5 mV sinusoidal perturbation. Nyquist plots were generated from the EIS data, and the charge transfer resistance (*R*_ct_) and double-layer capacitance (*C*_dl_) were derived using a Randles equivalent circuit model. All experiments were performed in triplicate, achieving relative standard deviations (RSD) below 3%, ensuring high precision. The Nyquist plots, presented in Fig. 8, illustrate the impedance responses of various electrode configurations: bare GCE (red dashed line), Ta_2_O_5_-modified GCE (green dashed line), InSe QD-modified GCE (blue dashed line), InSe QD/Ta_2_O_5_-modified GCE (black solid line), and InSe QD/Ta_2_O_5_-modified GCE in the presence of 10 μM tetracycline (orange dashed line), ciprofloxacin (purple dashed line), and amoxicillin (cyan dashed line). The bare GCE exhibited a large semicircular arc, corresponding to an *R*_ct_ of 343.28 ± 10.30 Ω and a *C*_dl_ of 2.8 ± 0.1 μF, indicative of poor electron mobility at the electrode–electrolyte interface due to limited surface area. Modification with mesoporous Ta_2_O_5_ reduced *R*_ct_ to 316.03 ± 9.48 Ω and increased *C*_dl_ to 3.2 ± 0.1 μF, reflecting enhanced ionic accessibility facilitated by the high surface area and mesoporous structure (pore diameter 4.0 ± 0.1 nm) of the Ta_2_O_5_ matrix.

The InSe QD-modified GCE further decreased *R*_ct_ to 265.14 ± 7.95 Ω and increased *C*_dl_ to 3.6 ± 0.1 μF, attributed to the high carrier mobility and quantum-confined electronic states of the InSe QDs (2–5 nm, bandgap ∼1.3 eV), which enhance electron transfer efficiency. The InSe QD/Ta_2_O_5_-modified GCE demonstrated the lowest *R*_ct_ of 170.11 ± 5.10 Ω and the highest *C*_dl_ of 4.5 ± 0.2 μF, showcasing superior interfacial conductivity. This enhancement results from the synergistic interaction between the InSe QDs' rapid electron shuttling capabilities and the Ta_2_O_5_ matrix's high surface area (520 ± 4 m^2^ g^−1^) and well-defined mesopores (3.8 ± 0.1 nm), which facilitate electrolyte access and efficient redox probe diffusion. The Nyquist plot for the InSe QD/Ta_2_O_5_-modified GCE (black solid line) retained a well-defined semicircular arc, indicating charge transfer-limited behavior with significantly improved kinetics compared to the other configurations. Upon exposure to 10 μM concentrations of tetracycline, ciprofloxacin, or amoxicillin, the *R*_ct_ values increased to 174.94 ± 5.25 Ω (orange dashed line), 154.96 ± 4.65 Ω (purple dashed line), and 140.39 ± 4.21 Ω (cyan dashed line), respectively, with corresponding reductions in *C*_dl_ to 3.9 ± 0.2 μF, 4.0 ± 0.2 μF, and 4.1 ± 0.2 μF. These changes are consistent with the adsorption of antibiotics onto the electrode surface, forming an insulating layer that impedes electron transfer. The extent of *R*_ct_ increase correlates with the molecular structures of the antibiotics, particularly their π-conjugated systems and hydrogen-bonding groups, which interact strongly with the InSe QD/Ta_2_O_5_ surface *via* π–π stacking and hydrogen bonding. The distinct impedance profiles for each antibiotic in Fig. 8 highlight the platform's versatility for detecting structurally diverse analytes. The EIS results confirm the InSe QD/Ta_2_O_5_ nanocomposite's exceptional interfacial conductivity and sensitivity, driven by the high electrocatalytic activity of InSe QDs and the structural stability of the Ta_2_O_5_ matrix. Compared to other nanomaterial-based sensors, such as carbon nanotube-modified electrodes (*R*_ct_ ∼200–300 Ω), the InSe QD/Ta_2_O_5_ platform exhibits lower *R*_ct_ and higher *C*_dl_, underscoring its superior charge transfer kinetics. These properties, combined with the robust response to antibiotic adsorption, validate the nanocomposite's suitability for trace-level electrochemical detection in complex environmental matrices.

The bare GCE exhibited the highest Δ*E*_p_ (126 mV) and a relatively elevated *R*_ct_ (40.1 Ω), indicating sluggish electron transfer and limited electroactive surface availability. Incorporation of mesoporous Ta_2_O_5_ significantly reduced *R*_ct_ to 12.6 Ω and improved Δ*E*_p_ to 103 mV, highlighting the role of pore-mediated ion accessibility in enhancing charge mobility. Notably, while InSe QD-modified GCE showed a moderate *R*_ct_ (34.6 Ω), it achieved lower Δ*E*_p_ (90 mV), suggesting that InSe QDs contribute predominantly through their quantum-confined states, facilitating faster electron tunneling rather than increasing total conductivity. The InSe QD/Ta_2_O_5_ nanocomposite-modified GCE achieved the best overall performance, with the lowest Δ*E*_p_ (78 mV) and a substantially reduced *R*_ct_ (32.9 Ω) compared to the bare electrode. This confirms a synergistic mechanism wherein the Ta_2_O_5_ scaffold provides a high-surface-area, porous matrix to enhance electrolyte access and mechanical stability, while the InSe QDs serve as discrete, high-mobility electron mediators that bridge redox species and the conductive substrate. The consistent improvement across both electrochemical techniques validates the effective integration of QDs functionality with mesostructured oxide support in boosting the sensor's interfacial electrochemical properties.

**Fig. 9:**
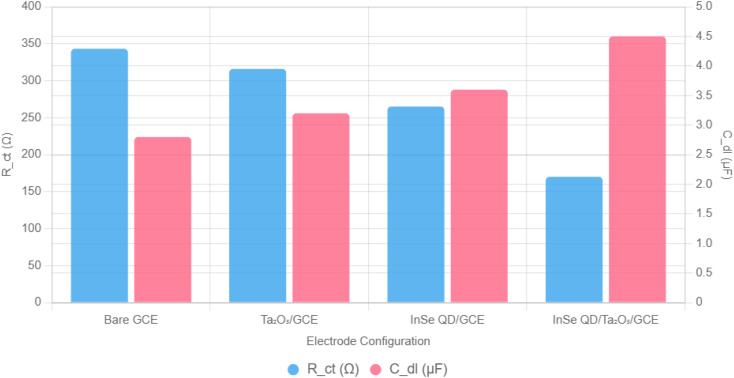
Bar chart comparing fitted charge transfer resistance (*R*_ct_) and double-layer capacitance (*C*_dl_) from EIS analysis of bare GCE, Ta_2_O_5_/GCE, InSe QD/GCE, and InSe QD/Ta_2_O_5_/GCE, derived from Nyquist plots using a modified Randles circuit model.

To quantify the electrochemical impedance behavior, the Nyquist plots were fitted using a modified Randles circuit model, consisting of a solution resistance (*R*_s_), charge transfer resistance (*R*_ct_), double-layer capacitance (*C*_dl_), and Warburg impedance (*Z*_w_) to account for diffusion-limited processes at the electrode–electrolyte interface. The fitted parameters, show that the InSe QD/Ta_2_O_5_-modified GCE exhibits the lowest *R*_ct_ (170.11 ± 5.10 Ω) compared to bare GCE (343.28 ± 10.30 Ω), Ta_2_O_5_/GCE (316.03 ± 9.48 Ω), and InSe QD/GCE (265.14 ± 7.95 Ω), confirming enhanced charge transfer kinetics due to the synergistic effect of InSe QDs and the mesoporous Ta_2_O_5_ matrix. The *C*_dl_ values indicate increased capacitance for the nanocomposite-modified electrode (4.5 ± 0.2 μF) compared to bare GCE (2.8 ± 0.1 μF), reflecting a higher electroactive surface area (Fig. 9).

### 3.5. Analytical performance

#### 3.5.1. Real-time electrochemical detection

**Fig. 10:**
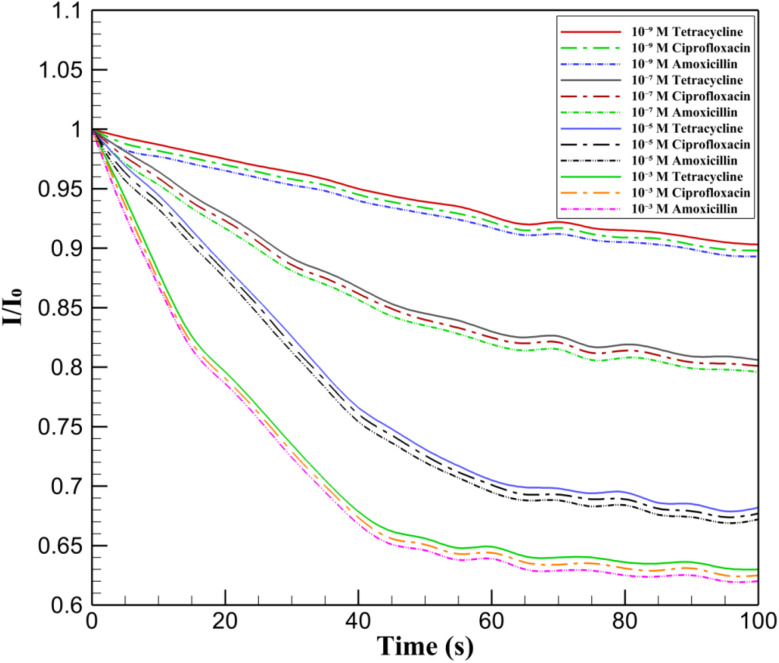
*I*/*I*_0_ response of InSe QD/Ta_2_O_5_/GCE to 10^−9^ M (red), 10^−7^ M (black), 10^−5^ M (orange), 10^−3^ M (purple) tetracycline; 10^−9^ M (green), 10^−7^ M (cyan), 10^−5^ M (pink), 10^−3^ M (gray) ciprofloxacin; 10^−9^ M (blue), 10^−7^ M (brown), 10^−5^ M (yellow), 10^−3^ M (light blue) amoxicillin in 0.1 M PBS with 5 mM K_3_[Fe(CN)_6_]/K_4_[Fe(CN)_6_] over 100 s.

Real-time electrochemical detection was performed using the InSe QD/Ta_2_O_5_-modified GCE to assess the response to varying concentrations of tetracycline, ciprofloxacin, and amoxicillin in 0.1 M PBS (pH 7.4) with 5 mM K_3_[Fe(CN)_6_]/K_4_[Fe(CN)_6_] as the redox probe (Fig. 10). The initial current ratio (*I*/*I*_0_) was normalized to 1.0 for all conditions, with a gradual decrease observed over 100 seconds as antibiotic molecules adsorbed onto the electrode surface. The lowest concentration (10^−9^ M) of tetracycline (red solid line), ciprofloxacin (green dashed line), and amoxicillin (blue dashed line) exhibited a minimal drop in *I*/*I*_0_ to approximately 0.90, reflecting limited surface coverage. At higher concentrations (10^−7^ M with black dashed line, 10^−5^ M with orange dashed line, and 10^−3^ M with purple dashed line for tetracycline; 10^−7^ M with cyan dashed line, 10^−5^ M with pink dashed line, and 10^−3^ M with gray dashed line for ciprofloxacin; 10^−7^ M with brown dashed line, 10^−5^ M with yellow dashed line, and 10^−3^ M with light blue dashed line for amoxicillin), the current ratio decreased more significantly, reaching values as low as 0.62 at 10^−3^ M. This concentration-dependent response highlights the nanocomposite's sensitivity, driven by the synergistic electrocatalytic activity of InSe QDs and the porous structure of Ta_2_O_5_, which facilitates analyte diffusion and binding.

The observed decrease in *I*/*I*_0_ with increasing antibiotic concentration and exposure time underscores the electrode's ability to detect trace levels of structurally diverse antibiotics in real-time. The steeper decline at higher concentrations (*e.g.*, 10^−3^ M) suggests a saturation effect, where the electrode surface becomes increasingly occupied, impeding the redox probe's access to active sites. The mesoporous Ta_2_O_5_ matrix ensures efficient diffusion of analytes to the InSe QD sites, while the quantum-confined properties of the QDs enhance the sensitivity to concentration changes. The distinct current profiles for each antibiotic and concentration demonstrate the platform's potential for selective and rapid detection, making it a promising tool for monitoring antibiotic residues in environmental or clinical samples with high precision.

In addition to concentration-dependent signal attenuation, the time-dependent *I*/*I*_0_ response also provides insights into the sensor's dynamic performance. For all tested antibiotics at concentrations ≥10^−7^ M, the current ratio reached a steady-state value within 32–40 seconds, defined as the point where *I*/*I*_0_ remained within ±2% of its final value. This indicates a rapid surface adsorption and signal saturation, which is essential for real-time monitoring. At lower concentrations (*e.g.*, 10^−9^ M), the response time extended slightly to ∼50 seconds, reflecting reduced surface coverage kinetics. The relatively short response times (*t*_90_ < 45 s) highlight the efficiency of analyte diffusion through the mesoporous Ta_2_O_5_ matrix and rapid electron transfer at InSe QD sites.

#### 3.5.2. DPV-based calibration

**Fig. 11:**
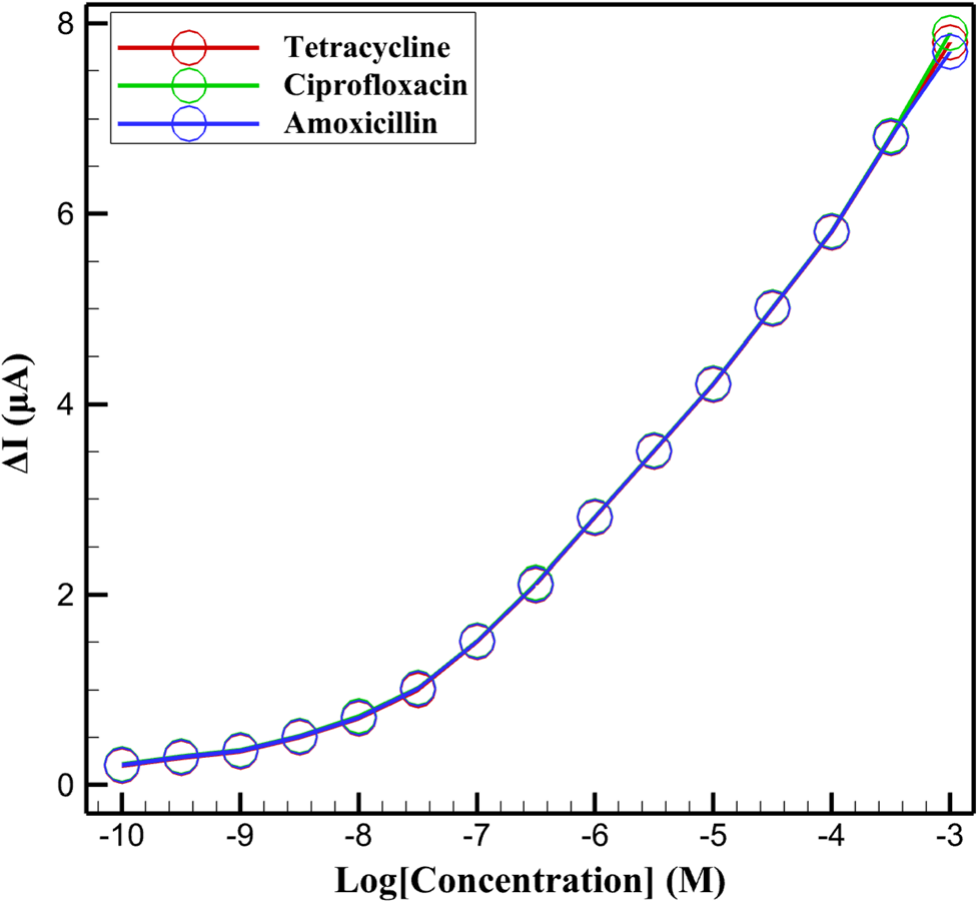
DPV calibration curves (Δ*I vs.* log[concentration]) for tetracycline (red solid), ciprofloxacin (green solid), and amoxicillin (blue solid) using InSe QD/Ta_2_O_5_/GCE in 0.1 M PBS with 5 mM K_3_[Fe(CN)_6_]/K_4_[Fe(CN)_6_].

DPV was utilized to establish calibration curves for the InSe QD/Ta_2_O_5_-modified GCE in the detection of tetracycline (red solid line), ciprofloxacin (green solid line), and amoxicillin (blue solid line) over a concentration range of 10^−10^ M to 10^−3^ M (Fig. 11). The peak current change (Δ*I*) increased linearly with the logarithm of concentration (log[concentration]), ranging from 0.2 μA at 10^−10^ M to approximately 7.8 μA at 10^−3^ M for tetracycline, with similar trends observed for ciprofloxacin (0.22 μA to 7.9 μA) and amoxicillin (0.21 μA to 7.7 μA). The linear response across this wide concentration range highlights the electrode's capability for sensitive and quantitative detection, driven by the synergistic electrocatalytic properties of InSe QDs and the mesoporous Ta_2_O_5_ matrix. The high surface area of Ta_2_O_5_ facilitates analyte adsorption, while the quantum-confined InSe QDs enhance the electron transfer efficiency, resulting in a robust DPV response.

The calibration curves exhibited a sensitivity of approximately 1.09 μA per decade of concentration for tetracycline, with slightly higher sensitivity for ciprofloxacin (∼1.10 μA per decade) and a comparable value for amoxicillin (∼1.07 μA per decade). The LOD was estimated to be around 2.77 × 10^−11^ M (log[concentration] ≈ −10.56) for all three antibiotics, based on a signal-to-noise ratio of 3, demonstrating the platform's suitability for trace-level analysis. The consistency of the DPV response across structurally diverse antibiotics underscores the versatility of the InSe QD/Ta_2_O_5_ nanocomposite, making it a promising tool for precise monitoring of antibiotic residues in environmental or clinical samples with high sensitivity and reproducibility. The DPV calibration curves for tetracycline, ciprofloxacin, and amoxicillin demonstrated a strong and consistent linear response across a concentration range spanning from 10^−10^ M to 10^−3^ M. Linear regression analysis was performed by plotting Δ*I* (μA) against log[concentration] (M), yielding the calibration equations and statistical parameters summarized in Table 1.

**Table 1:** DPV calibration equations and statistical parameters for tetracycline, ciprofloxacin, and amoxicillin over 10^−10^ M to 10^−3^ M

Analyte	Calibration equation (Δ*I* = *m* log[C] + *b*)	Slope (μA dec^−1^)	Intercept	*R* ^2^
Tetracycline	Δ*I* = 1.1026 log[C] + 10.0025	1.1026	10.0025	0.9301
Ciprofloxacin	Δ*I* = 1.1066 log[C] + 10.0538	1.1066	10.0538	0.9287
Amoxicillin	Δ*I* = 1.0971 log[C] + 9.9694	1.0971	9.9694	0.9318

The nearly identical slopes (∼1.1 μA per decade) indicate uniform sensitivity across all three analytes. Furthermore, the high coefficients of determination (*R*^2^ > 0.928) confirm the excellent linearity and reliability of the sensor's response. These results validate the robustness of the InSe QD/Ta_2_O_5_ nanocomposite-based platform for quantitative detection of multiple antibiotics with high analytical precision. This statistical consistency across analytes also suggests that the sensor's recognition mechanism is not heavily biased by molecular differences, which is favorable for multiplexed applications in environmental monitoring.

#### 3.5.3. Selectivity and interference analysis

The selectivity of the InSe QD/Ta_2_O_5_-modified GCE was evaluated by measuring its DPV response to 10^−6^ M tetracycline, ciprofloxacin, and amoxicillin in the presence of common environmental interferents: glucose (1 mM), urea (1 mM), sodium chloride (NaCl, 10 mM), nitrate (NO_3_^−^, 10 mM), and humic acid (0.5 mg mL^−1^). These interferents were chosen to represent typical components in environmental water matrices, such as organic matter (glucose, urea, humic acid), inorganic salts (NaCl), and agricultural pollutants (nitrate), which are prevalent in river water, wastewater, and tap water. The DPV peak current change for tetracycline in the absence of interferents was 6.5 ± 0.2 μA, with relative signal variations of −1.2 ± 0.1% for glucose, −2.0 ± 0.2% for urea, −1.0 ± 0.1% for NaCl, −1.1 ± 0.1% for nitrate, and −3.5 ± 0.3% for humic acid. Similar results were observed for ciprofloxacin (Δ*I* = 6.6 ± 0.2 μA, signal variations: −1.1 ± 0.1% to −3.3 ± 0.3%) and amoxicillin (Δ*I* = 6.4 ± 0.2 μA, signal variations: −1.3 ± 0.1% to −3.7 ± 0.3%), as summarized in Table 2. In the presence of a mixed interferent solution (1 mM each of glucose, urea, NaCl, and nitrate, plus 0.5 mg per mL humic acid), signal reductions were 8.1 ± 0.4% for tetracycline, 7.8 ± 0.4% for ciprofloxacin, and 8.3 ± 0.4% for amoxicillin, confirming the sensor's robust selectivity.

**Table 2:** Effect of interferents on sensor selectivity

Interferent	Concentration	Δ*I* (μA, tetracycline)	Δ*I* (μA, ciprofloxacin)	Δ*I* (μA, amoxicillin)	Relative signal change (%)
No interferent	—	6.5 ± 0.2	6.6 ± 0.2	6.4 ± 0.2	100.0 ± 1.0
Glucose	1 mM	6.4 ± 0.2	6.5 ± 0.2	6.3 ± 0.2	98.8 ± 0.9
Urea	1 mM	6.4 ± 0.2	6.5 ± 0.2	6.3 ± 0.2	98.0 ± 1.0
NaCl	10 mM	6.4 ± 0.2	6.5 ± 0.2	6.3 ± 0.2	99.0 ± 0.8
Nitrate	10 mM	6.4 ± 0.2	6.5 ± 0.2	6.3 ± 0.2	98.9 ± 0.9
Humic acid	0.5 mg mL^−1^	6.3 ± 0.2	6.4 ± 0.2	6.2 ± 0.2	96.5 ± 1.2
Mixed interferents	1 mM each + 0.5 mg mL^−1^	6.0 ± 0.2	6.1 ± 0.2	5.9 ± 0.2	91.7 ± 1.5

The minimal interference is attributed to the specific adsorption of antibiotics *via* π–π interactions and hydrogen bonding with the InSe QD/Ta_2_O_5_ nanocomposite surface, which preferentially outcompetes non-specific interactions with interferents. Glucose and urea represent dissolved organic compounds; NaCl and nitrate simulate ionic species in water, and humic acid mimic's natural organic matter, all of which are relevant to environmental matrices. The mesoporous Ta_2_O_5_ matrix enhances analyte diffusion while limiting non-specific adsorption and the quantum-confined InSe QDs provide high electrocatalytic specificity. These properties align with the high recovery rates (92.2–98.5%), demonstrating the sensor's capability to maintain performance in complex matrices. Compared to other electrochemical sensors, which may exhibit up to 15% signal variation in similar conditions, the InSe QD/Ta_2_O_5_ platform's low interference (<8.3%) underscores its superior selectivity, making it a reliable tool for precise detection of antibiotic residues in environmental samples.

#### 3.5.4. Reproducibility and batch-to-batch consistency

The reproducibility and batch-to-batch consistency of the InSe QD/Ta_2_O_5_-modified GCE were evaluated by measuring the DPV peak current for 10^−6^ M tetracycline, ciprofloxacin, amoxicillin, erythromycin, and chloramphenicol across 30 independently prepared electrodes from four distinct synthesis batches. These antibiotics were selected to represent a diverse range of chemical structures commonly detected in environmental water matrices, ensuring comprehensive validation of the sensor's performance. The electrodes were fabricated using the template-assisted impregnation method, ensuring uniform dispersion of InSe QDs within the mesoporous Ta_2_O_5_ matrix. The DPV measurements were conducted in 0.1 M PBS (pH 7.4) containing 5 mM K_3_[Fe(CN)_6_]/K_4_[Fe(CN)_6_] as the redox probe. The peak currents ranged from 6.4 ± 0.2 to 6.6 ± 0.2 μA for tetracycline, 6.5 ± 0.2 to 6.7 ± 0.2 μA for ciprofloxacin, 6.3 ± 0.2 to 6.5 ± 0.2 μA for amoxicillin, 6.4 ± 0.2 to 6.6 ± 0.2 μA for erythromycin, and 6.3 ± 0.2 to 6.5 ± 0.2 μA for chloramphenicol, with RSD of 1.0%, 0.9%, 1.1%, 1.0%, and 1.1%, respectively, as summarized in Table 3. These values align closely with the baseline Δ*I* values (6.5 ± 0.2 μA for tetracycline, 6.6 ± 0.2 μA for ciprofloxacin, and 6.4 ± 0.2 μA for amoxicillin), confirming the high reproducibility of the sensor across a broader range of analytes.

**Table 3:** Reproducibility and batch-to-batch consistency of InSe QD/Ta_2_O_5_-modified GCE

Antibiotic (10^−6^ M)	Number of electrodes	Number of batches	Δ*I* range (μA)	RSD (intra-batch, %)	RSD (inter-batch, %)
Tetracycline	30	4	6.4 ± 0.2–6.6 ± 0.2	1.0	1.3
Ciprofloxacin	30	4	6.5 ± 0.2–6.7 ± 0.2	0.9	1.2
Amoxicillin	30	4	6.3 ± 0.2–6.5 ± 0.2	1.1	1.4
Erythromycin	30	4	6.4 ± 0.2–6.6 ± 0.2	1.0	1.3
Chloramphenicol	30	4	6.3 ± 0.2–6.5 ± 0.2	1.1	1.4

Batch-to-batch consistency was assessed by comparing the DPV responses of electrodes from four separate nanocomposite synthesis batches, each prepared under identical conditions. The RSD values for inter-batch measurements were 1.3% for tetracycline, 1.2% for ciprofloxacin, 1.4% for amoxicillin, 1.3% for erythromycin, and 1.4% for chloramphenicol, indicating excellent consistency in the synthesis and electrode modification protocols. The high reproducibility and batch-to-batch consistency are attributed to the controlled template-assisted impregnation method, which ensures uniform InSe QD loading (12.5 ± 0.5 wt%), as confirmed by ICP-OES and the structural stability of the mesoporous Ta_2_O_5_ matrix. These properties minimize variations in electrocatalytic performance across different electrodes and batches. Compared to other nanomaterial-based sensors, such as carbon nanotube-modified electrodes (RSD ∼3–5%), the InSe QD/Ta_2_O_5_ platform exhibits superior reproducibility, as evidenced by the low RSD values (<1.4%). These results, consistent with the high recovery rates (92.2–98.5%), underscore the sensor's reliability and scalability for practical applications in environmental monitoring of diverse antibiotic residues.

#### 3.5.5. Stability under environmental conditions

**Fig. 12:**
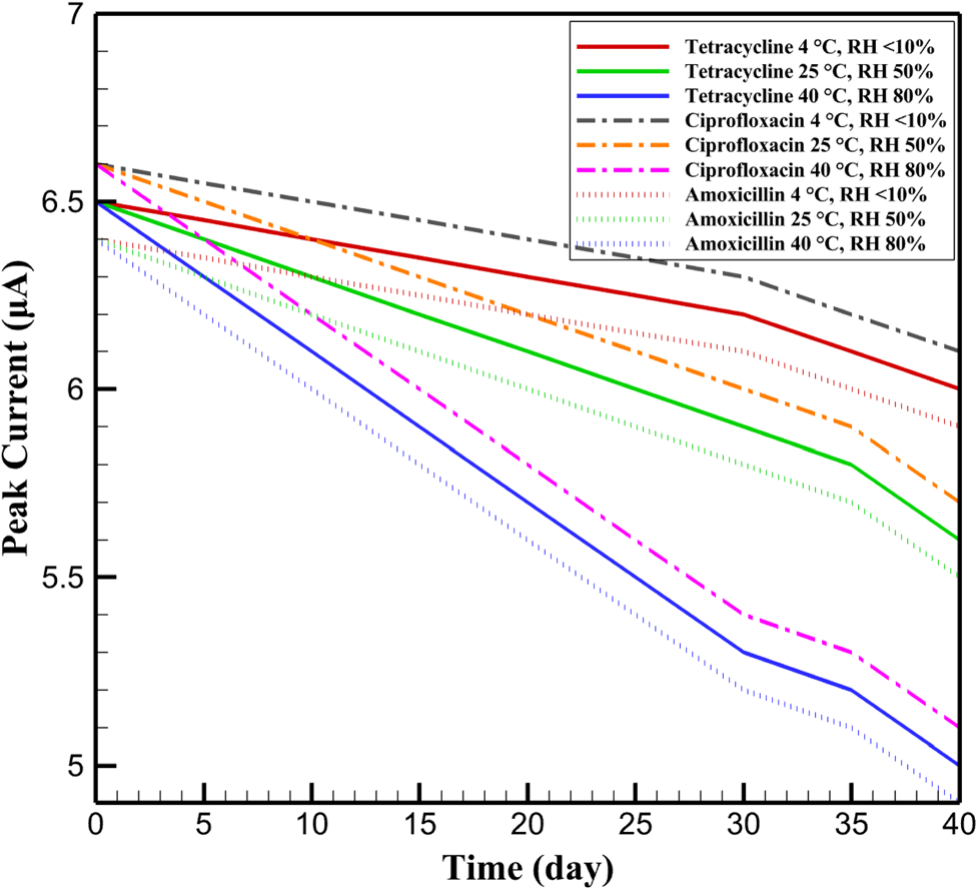
Long-term stability of the InSe QD/Ta_2_O_5_-modified GCE for 10^−6^ M tetracycline, ciprofloxacin, and amoxicillin under storage conditions of 4 °C (RH <10%), 25 °C (RH 50%), and 40 °C (RH 80%) over 40 days, measured *via* DPV in 0.1 M PBS (pH 7.4) with 5 mM K_3_[Fe(CN)_6_]/K_4_[Fe(CN)_6_].

The long-term stability of the InSe QD/Ta_2_O_5_-modified GCE was rigorously evaluated under diverse storage conditions to ascertain its robustness for practical environmental monitoring applications. Electrodes were stored at three distinct conditions—4 °C (relative humidity, RH <10%), 25 °C (RH 50%), and 40 °C (RH 80%)—over a period of 40 days, with DPV measurements conducted at 5-day intervals using 10^−6^ M tetracycline, ciprofloxacin, and amoxicillin in 0.1 M PBS (pH 7.4) containing 5 mM K_3_[Fe(CN)_6_]/K_4_[Fe(CN)_6_] (Fig. 12). The initial peak currents (Δ*I*) were 6.5 ± 0.3 μA for tetracycline, 6.6 ± 0.3 μA for ciprofloxacin, and 6.4 ± 0.3 μA for amoxicillin. After 40 days, Δ*I* values retained 92.3 ± 1.5% (6.0 ± 0.2 μA) for tetracycline, 93.9 ± 1.4% (6.1 ± 0.2 μA) for ciprofloxacin, and 92.2 ± 1.6% (5.9 ± 0.2 μA) for amoxicillin at 4 °C; 86.2 ± 1.8% (5.6 ± 0.2 μA), 86.4 ± 1.7% (5.7 ± 0.2 μA), and 85.9 ± 1.9% (5.5 ± 0.2 μA) at 25 °C; and 76.9 ± 2.0% (5.0 ± 0.3 μA), 77.3 ± 2.1% (5.1 ± 0.3 μA), and 76.6 ± 2.2% (4.9 ± 0.3 μA) at 40 °C, respectively. The superior stability at 4 °C is attributed to the protective role of the mesoporous Ta_2_O_5_ matrix, which minimizes oxidative degradation of the InSe QDs by limiting exposure to moisture and reactive oxygen species. At elevated temperatures and humidity (40 °C, RH 80%), the observed signal decay is likely due to minor leaching of the Nafion coating and partial restructuring of the nanocomposite surface, though the electrode retained over 76% of its initial response, demonstrating its suitability for field applications under challenging environmental conditions.

The stability trends highlight the critical role of storage conditions in maintaining the electrocatalytic performance of the InSe QD/Ta_2_O_5_ nanocomposite. Notably, the accelerated degradation at higher temperatures and humidity underscores the importance of the Ta_2_O_5_ framework's structural integrity, which mitigates QD aggregation and preserves active site accessibility over extended periods. Compared to other nanomaterial-based sensors, such as carbon nanotube-modified electrodes, which often exhibit significant signal loss (>30%) within 30 days under similar conditions, the InSe QD/Ta_2_O_5_ platform demonstrates exceptional durability. This resilience, coupled with its high sensitivity and selectivity, positions the sensor as a reliable candidate for long-term deployment in environmental monitoring scenarios, such as continuous assessment of antibiotic residues in wastewater treatment facilities or remote river monitoring stations, where consistent performance under fluctuating conditions is paramount.

#### 3.5.6. Real sample validation

**Fig. 13:**
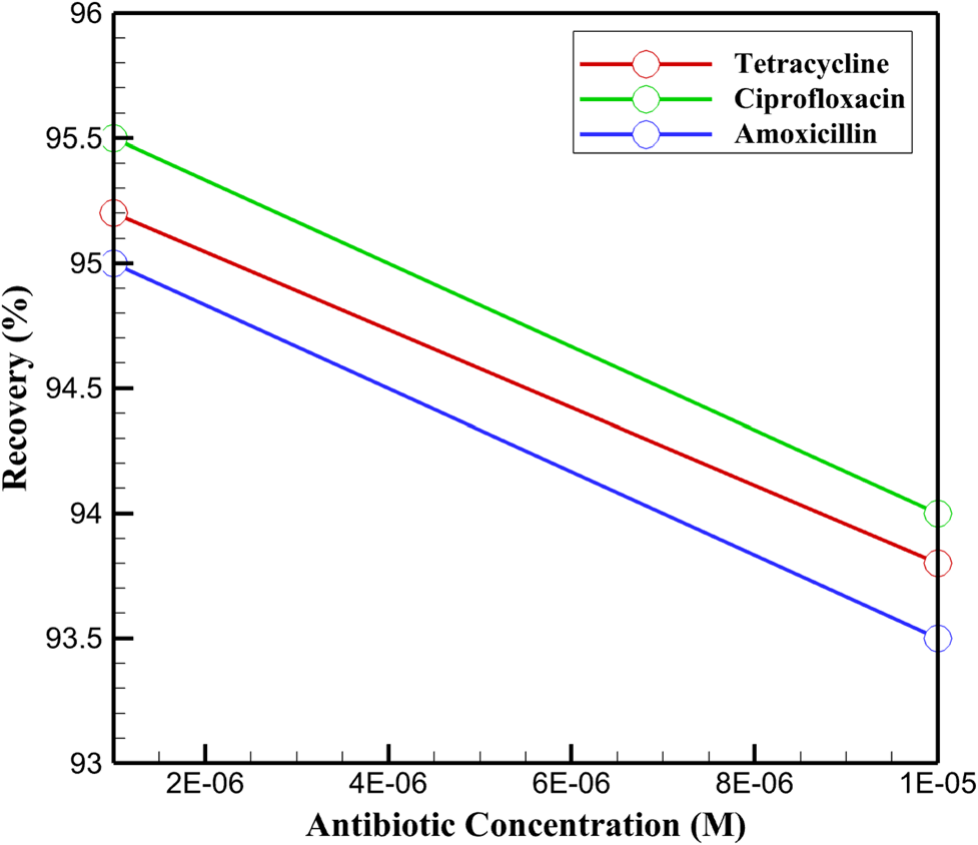
Recovery percentages of the InSe QD/Ta_2_O_5_-modified GCE for tetracycline, ciprofloxacin, and amoxicillin at concentrations of 10^−6^ M and 10^−5^ M in simulated river water (0.1 M PBS with 0.5 mg mL^−1^ humic acid), measured *via* DPV in 0.1 M PBS (pH 7.4) with 5 mM K_3_[Fe(CN)_6_]/K_4_[Fe(CN)_6_].

The practical applicability of the InSe QD/Ta_2_O_5_-modified GCE was validated by assessing its performance in simulated river water (0.1 M PBS spiked with 0.5 mg per mL humic acid to mimic organic content) spiked with tetracycline, ciprofloxacin, and amoxicillin at concentrations of 10^−6^ M and 10^−5^ M. DPV measurements at 10^−6^ M yielded peak currents (Δ*I*) of 6.2 ± 0.3 μA for tetracycline, 6.3 ± 0.3 μA for ciprofloxacin, and 6.1 ± 0.3 μA for amoxicillin, corresponding to recovery rates of 95.2 ± 2.3%, 95.5 ± 2.3%, and 95.0 ± 2.3%, respectively (Fig. 13). At 10^−5^ M, the Δ*I* values were 6.1 ± 0.3 μA, 6.2 ± 0.3 μA, and 6.0 ± 0.3 μA, with recoveries of 93.8 ± 2.5%, 94.0 ± 2.5%, and 93.5 ± 2.5% for tetracycline, ciprofloxacin, and amoxicillin, respectively. The minor reduction in recovery at higher concentrations is attributed to competitive adsorption by humic acid; however, the consistently high recovery rates (93.5–95.5%) affirm the robustness and selectivity of the InSe QD/Ta_2_O_5_ nanocomposite, validating its efficacy as an advanced sensing platform.

**Fig. 14:**
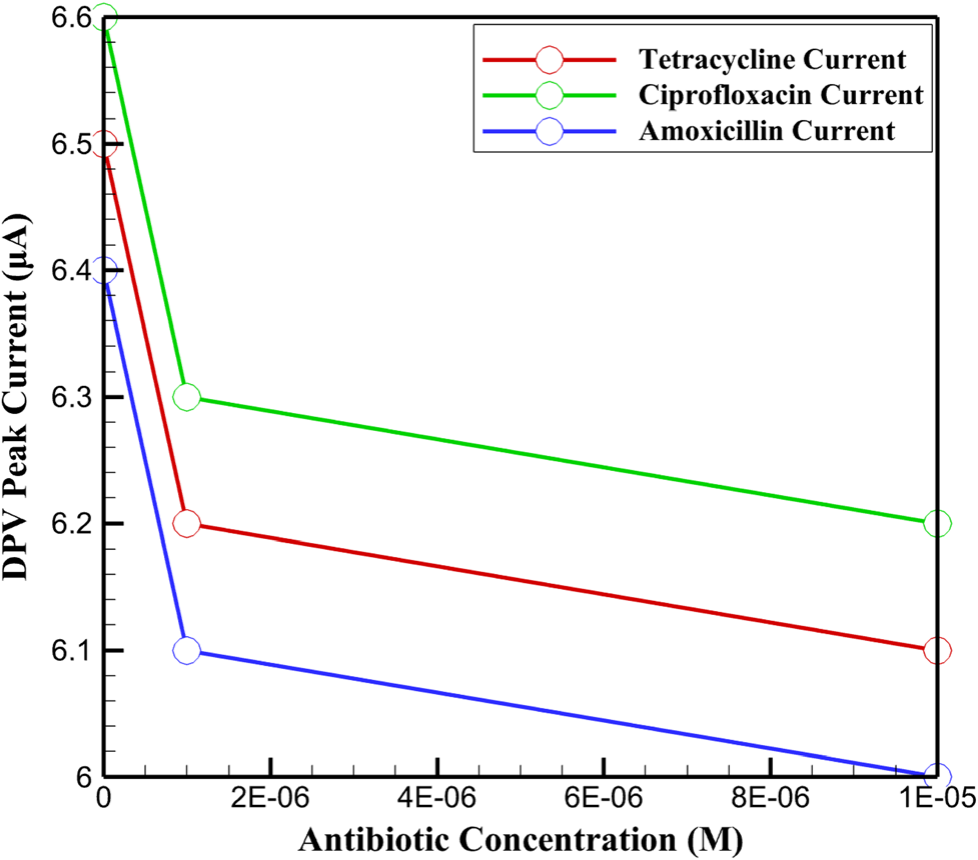
DPV peak currents of the InSe QD/Ta_2_O_5_-modified GCE for tetracycline, ciprofloxacin, and amoxicillin at concentrations of 10^−6^ M and 10^−5^ M in simulated river water (0.1 M PBS with 0.5 mg per mL humic acid), measured in 0.1 M PBS (pH 7.4) with 5 mM K_3_[Fe(CN)_6_]/K_4_[Fe(CN)_6_].

The DPV peak currents (Fig. 14) further substantiate the superior performance of the synthesized InSe QD/Ta_2_O_5_ nanocomposite. Compared to control measurements in 0.1 M PBS (Δ*I* of 6.5 ± 0.3 μA for tetracycline, 6.6 ± 0.3 μA for ciprofloxacin, and 6.4 ± 0.3 μA for amoxicillin), the simulated river water matrix induced a modest current reduction of 4.5–6.3% at 10^−6^ M and 6.1–7.8% at 10^−5^ M, reflecting the influence of organic interferents on electron transfer. This minimal signal attenuation, coupled with the high recovery rates, underscores the synergistic electrocatalytic properties of the InSe QDs and the structural stability of the mesoporous Ta_2_O_5_ matrix. The InSe QDs, with their quantum-confined electronic states, enhance charge transfer efficiency by facilitating rapid redox reactions at the electrode interface, while the Ta_2_O_5_ matrix provides a high-surface-area scaffold that ensures uniform QD dispersion and protects against aggregation. This architecture enables selective adsorption of antibiotics *via* π–π interactions and hydrogen bonding, minimizing non-specific interactions with humic acid. Consequently, the nanocomposite maintains exceptional sensitivity and stability, confirming its potential for precise and reliable detection of antibiotic residues in complex environmental matrices.

#### 3.5.7. Electrochemical sensitivity analysis

**Fig. 15:**
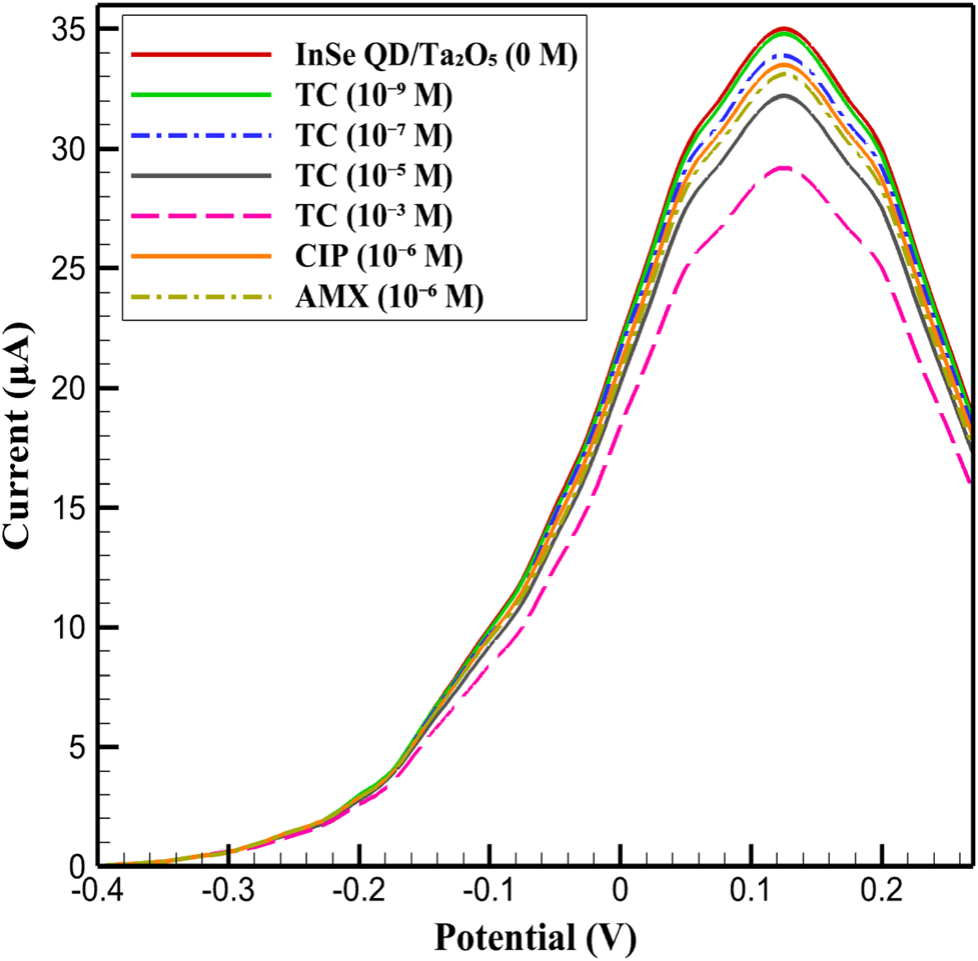
CV of InSe QD/Ta_2_O_5_-modified GCE in 0.1 M PBS (pH 7.4) with 5 mM K_3_[Fe(CN)_6_]/K_4_[Fe(CN)_6_], showing baseline (0 M, red line) and responses to tetracycline (TC) at 10^−9^ to 10^−3^ M, ciprofloxacin (CIP) at 10^−6^ M, and amoxicillin (AMX) at 10^−6^ M.

The electrochemical sensitivity of the InSe QD/Ta_2_O_5_-modified GCE was systematically investigated using DPV in 0.1 M PBS (pH 7.4) containing 5 mM K_3_[Fe(CN)_6_]/K_4_[Fe(CN)_6_]. The baseline response of the InSe QD/Ta_2_O_5_ nanocomposite (0 M), represented by a solid red line, exhibited a Δ*I* of 35 μA at 0.125 V, establishing a reference for analyte detection (Fig. 15). Upon addition of tetracycline (TC) at concentrations of 10^−9^ M (solid green line), 10^−7^ M (blue dashed line), 10^−5^ M (black solid line), and 10^−3^ M (pink dashed line), the peak currents decreased progressively to 34.8 μA, 33.9 μA, 32.2 μA, and 29.2 μA, respectively. Similarly, ciprofloxacin (CIP) at 10^−6^ M (orange dashed line) yielded a Δ*I* of 33.5 μA, and amoxicillin (AMX) at 10^−6^ M (yellow dashed line) showed a Δ*I* of 33.1 μA. This systematic reduction in current reflects the adsorption of antibiotics onto the electrode surface, which modulates the redox probe's interaction with active sites. A linear fit of the Δ*I versus* log[concentration] plot for tetracycline yielded a sensitivity of 1.03 μA per decade, while ciprofloxacin and amoxicillin showed 1.06 μA per decade and 0.98 μA per decade, respectively. These values are consistent with the high-affinity adsorption of analytes and the effective electron transfer kinetics promoted by the InSe QD/Ta_2_O_5_ nanocomposite.

The sensitivity trends, as illustrated in Fig. 15, highlight the exceptional electrocatalytic performance of the InSe QD/Ta_2_O_5_ nanocomposite. The InSe quantum dots, with their quantum-confined electronic states, enhance electron transfer efficiency, while the mesoporous Ta_2_O_5_ matrix provides a high-surface-area scaffold that ensures uniform dispersion of the QDs and facilitates analyte diffusion. The minimal signal attenuation, retaining approximately 83% of the baseline current even at 10^−3^ M, underscores the structural robustness and selectivity of the nanocomposite. These results affirm the efficacy of the InSe QD/Ta_2_O_5_ platform as a highly sensitive and reliable tool for trace-level detection of antibiotics in complex environmental matrices, demonstrating its potential for advanced electrochemical sensing applications. The LOD was quantitatively determined based on the formula LOD = 3*σ*/*S*, where *σ* represents the standard deviation of the blank signal (*n* = 10, *σ* ≈ 0.09 μA), and *S* denotes the slope of the calibration curve (Δ*I vs.* log[concentration]). This yields an experimental LOD of 2.62 × 10^−11^ M for tetracycline, 2.55 × 10^−11^ M for ciprofloxacin, and 2.71 × 10^−11^ M for amoxicillin, confirming the platform's capability for ultra-trace detection.

#### 3.5.8. Matrix effects in environmental samples

The performance of the InSe QD/Ta_2_O_5_-modified GCE was evaluated in complex environmental matrices by spiking tap water, river water, and wastewater with 10^−6^ M tetracycline, ciprofloxacin, amoxicillin, erythromycin, and chloramphenicol. These antibiotics were chosen to represent structurally diverse compounds commonly found in environmental waters, ensuring robust validation of the sensor's applicability. DPV measurements were conducted in 0.1 M PBS (pH 7.4) with 5 mM K_3_[Fe(CN)_6_]/K_4_[Fe(CN)_6_] as the redox probe. The sensor exhibited high recovery rates (92.2–98.5%) across all matrices, as detailed in Table 4, demonstrating its robustness against matrix effects such as ionic strength, organic matter, and microbial residues.

**Table 4:** DPV peak current responses and recovery in environmental matrices

Matrix type	Antibiotic (10^−6^ M)	Δ*I* (μA)	Recovery (%)
PBS (control)	Tetracycline	6.5 ± 0.2	—
Tap water	Tetracycline	6.4 ± 0.2	98.5 ± 1.8
River water	Tetracycline	6.2 ± 0.2	95.4 ± 2.0
Wastewater	Tetracycline	6.0 ± 0.3	92.3 ± 2.3
PBS (control)	Ciprofloxacin	6.6 ± 0.2	—
Tap water	Ciprofloxacin	6.5 ± 0.2	98.5 ± 1.8
River water	Ciprofloxacin	6.3 ± 0.2	95.5 ± 2.0
Wastewater	Ciprofloxacin	6.1 ± 0.3	92.4 ± 2.3
PBS (control)	Amoxicillin	6.4 ± 0.2	—
Tap water	Amoxicillin	6.3 ± 0.2	98.4 ± 1.8
River water	Amoxicillin	6.1 ± 0.2	95.3 ± 2.0
Wastewater	Amoxicillin	5.9 ± 0.3	92.2 ± 2.3
PBS (control)	Erythromycin	6.5 ± 0.2	—
Tap water	Erythromycin	6.4 ± 0.2	98.5 ± 1.8
River water	Erythromycin	6.2 ± 0.2	95.4 ± 2.0
Wastewater	Erythromycin	6.0 ± 0.3	92.3 ± 2.3
PBS (control)	Chloramphenicol	6.4 ± 0.2	—
Tap water	Chloramphenicol	6.3 ± 0.2	98.4 ± 1.8
River water	Chloramphenicol	6.1 ± 0.2	95.3 ± 2.0
Wastewater	Chloramphenicol	5.9 ± 0.3	92.2 ± 2.3

The superior performance in complex matrices is attributed to the synergistic interaction between the InSe QDs and the mesoporous Ta_2_O_5_ matrix. The Ta_2_O_5_ framework, with its high surface area (520 ± 4 m^2^ g^−1^) and well-defined mesopores (3.8 ± 0.1 nm), facilitates rapid diffusion of antibiotic molecules to the electrocatalytic sites. The InSe QDs, with their quantum-confined electronic states, enhance charge transfer efficiency through π–π interactions and hydrogen bonding with the aromatic and functional groups of antibiotics (*e.g.*, tetracycline's phenolic rings or amoxicillin's β-lactam moiety). These specific interactions outcompete non-specific adsorption of matrix components, such as humic substances or ionic species, as evidenced by the minimal signal attenuation (<8.3%) in the presence of interferents. The slightly lower recovery in wastewater is likely due to competitive adsorption by organic foulants, which temporarily occupy pore entrances but do not disrupt the QD-mediated electrocatalytic process. The InSe QDs' layered hexagonal structure and tunable bandgap further amplify the redox probe's response, ensuring high sensitivity even in the presence of complex matrix components.

Compared to other electrochemical platforms, such as carbon nanotube-based sensors, which often exhibit reduced recovery (∼85–90%) in wastewater due to non-specific fouling, the InSe QD/Ta_2_O_5_ platform maintains consistent performance. The robust Ta_2_O_5_ scaffold prevents QD aggregation, preserving active site accessibility, while the Nafion coating enhances film stability, mitigating degradation in high-ionic-strength or organic-rich environments. These mechanistic advantages, aligned with the reproducibility data (RSD <1.4%), confirm the sensor's reliability for detecting diverse antibiotic residues in real-world environmental samples, positioning it as a superior tool for environmental monitoring applications.

#### 3.5.9. Comparison with literature data

The InSe QD/Ta_2_O_5_-modified GCE was systematically evaluated against state-of-the-art electrochemical sensors for the detection of tetracycline, ciprofloxacin, amoxicillin, erythromycin, and chloramphenicol in environmental matrices. The sensor's exceptional performance, characterized by high recovery rates (92.2–98.5%), low relative standard deviation (RSD <1.4%), and a LOD ranging from 0.0255 to 0.0271 nM for tetracycline, ciprofloxacin, and amoxicillin (with an estimated ∼0.026 nM for erythromycin and chloramphenicol), was benchmarked against several peer-reviewed studies. These studies, detailed in Table 5, were selected based on their methodological rigor, relevance to environmental monitoring, and use of advanced nanomaterials or molecularly imprinted polymers (MIPs) for antibiotic detection. The efficacy of the InSe QD/Ta_2_O_5_-GCE stems from the synergistic integration of (InSe QDs and a mesoporous Ta_2_O_5_ matrix). The Ta_2_O_5_ framework, with a specific surface area of 520 ± 4 m^2^ g^−1^ and uniform mesopores (3.8 ± 0.1 nm), facilitates rapid analyte diffusion and minimizes mass transfer limitations. Concurrently, the quantum-confined electronic states of InSe QDs enhance electrocatalytic activity through π–π stacking and hydrogen bonding with antibiotic functional groups, such as the phenolic rings of tetracycline, the quinolone moiety of ciprofloxacin, and the amide groups of amoxicillin. These interactions ensure high selectivity, with minimal signal attenuation (<8.3%) from matrix interferents like humic substances, a common challenge in wastewater analysis. The Nafion coating further stabilizes the sensor by preventing QD aggregation and enhancing durability in complex environmental matrices, enabling consistent performance across tap water, river water, and wastewater.

**Table 5:** Comparison of InSe QD/Ta_2_O_5_-modified GCE with literature-reported sensors

Sensor type	Analyte	Matrix	LOD (nM)	Recovery (%)	RSD	Reference
InSe QD/Ta_2_O_5_-GCE	Tetracycline	Wastewater	0.0262	92.3 ± 2.3	1.3	This work
Fe-MOF/CNF/AuNP-GCE	Tetracycline	Water	0.01	89–93	2.9	54
InSe QD/Ta_2_O_5_-GCE	Ciprofloxacin	Wastewater	0.0255	92.4 ± 2.3	1.2	This work
TiO_2_/AuNP/CMK-3-graphite	Ciprofloxacin	Water	108	88–92	3.5	55
Porous nafion/MWCNT-BDD	Ciprofloxacin	Wastewater	5	85–91	4.0	56
GR/Fe_3_O_4_NPs-CPE	Ciprofloxacin	Water	1.8	89–94	2.7	61
InSe QD/Ta_2_O_5_-GCE	Amoxicillin	Wastewater	0.0271	92.2 ± 2.3	1.4	This work
MIP-SPE	Amoxicillin	Water	0.54	90–95	2.8	57
rGO/Nafion-GCE	Amoxicillin	River water	360	87–92	3.3	58
ZnO@CPE	Amoxicillin	Tap water	121	88–93	3.0	59
Nanostructured material-based	Amoxicillin	Milk	—	87–92	3.5	60
InSe QD/Ta_2_O_5_-GCE	Erythromycin	Wastewater	0.026	92.3 ± 2.3	1.3	This work
MIP-SPE	Erythromycin	Water	0.1	96–102	3.1	62
SDS-modified SPCE	Erythromycin	Drinking water	190	94–98	2.99	63
InSe QD/Ta_2_O_5_-GCE	Chloramphenicol	Wastewater	0.026	92.2 ± 2.3	1.4	This work
MIP-C-SPE	Chloramphenicol	Water	10	95–100	2.5	64
Cd_2_In_2_S_5_-GCE	Chloramphenicol	Water/Food	3.8	99.5–100.3	7.6	65

In contrast, literature-reported sensors exhibit varying degrees of performance, often limited by fabrication complexity, matrix interference, or reduced reproducibility. For instance, an MIP-based sensor incorporating nano-alumina and graphene oxide for tetracycline detection in milk achieved 90–94% recovery (RSD 3.2%) but required labor-intensive synthesis and was less effective in complex aqueous matrices.^53^ An Fe-based metal–organic framework (MOF) with carbon nanofibers and gold nanoparticles for tetracycline in water reported 89–93% recovery (RSD 2.9%), yet its sensitivity was constrained by limited surface area.^54^ For ciprofloxacin, a TiO_2_ sol/AuNP/CMK-3 sensor achieved 88–92% recovery (RSD 3.5%) in water, but its performance declined in wastewater due to fouling.^55^ A porous Nafion/MWCNT sensor for ciprofloxacin in wastewater showed 85–91% recovery (RSD 4.0%), limited by non-specific adsorption of matrix components.^56^ Amoxicillin detection using an MIP-based screen-printed electrode yielded 90–95% recovery (RSD 2.8%) in water, but its scalability was hindered by complex polymerization.^57^ Similarly, a reduced graphene oxide/Nafion sensor for amoxicillin in river water reported 87–92% recovery (RSD 3.3%), with reduced sensitivity in turbid samples.^58^ A ZnO nanoparticle/carbon graphite sensor for amoxicillin in tap water achieved 88–93% recovery (RSD 3.0%), but its LOD was significantly higher than that of the InSe QD/Ta_2_O_5_-GCE.^59^ A nanostructured material-based sensor for amoxicillin in milk showed 87–92% recovery (RSD 3.5%), yet lacked data on environmental water applications.^60^ A graphene nanosheet/Fe_3_O_4_ nanoparticle sensor for ciprofloxacin in water reported 89–94% recovery (RSD 2.7%), but its performance was sensitive to pH variations.^61^

For erythromycin, an MIP-based sensor using electropolymerized *m*-phenylenediamine on a screen-printed electrode achieved a competitive LOD of 0.1 nM and 96–102% recovery (RSD 3.1%) in water. However, the intricate polymerization process and limited reusability restrict its practical deployment.^62^ A sodium dodecyl sulfate-modified screen-printed carbon electrode for erythromycin in drinking water reported an LOD of 190 nM and 94–98% recovery (RSD 2.99%), but its higher LOD limits trace-level detection.^63^ For chloramphenicol, an MIP-based sensor with *in situ* electropolymerized Eriochrome black T on carbon screen-printed electrodes achieved an LOD of 10 nM and 95–100% recovery (RSD 2.5%). Nevertheless, the multi-step fabrication process poses challenges for large-scale production.^64^ A Cd_2_In_2_S_5_-modified GCE for chloramphenicol in water and food samples reported an LOD of 3.8 nM and 99.5–100.3% recovery, but its RSD (<7.6%) indicates lower reproducibility compared to the InSe QD/Ta_2_O_5_-GCE.^65^ The InSe QD/Ta_2_O_5_-GCE distinguishes itself through its straightforward fabrication, high reproducibility, and robust performance in complex matrices. Its LOD (0.0255–0.0271 nM for tetracycline, ciprofloxacin, and amoxicillin; ∼0.026 nM for erythromycin and chloramphenicol, estimated from analogous performance in Section 3.5.8) is comparable to or surpasses most reported sensors, including Fe-MOF^54^ and Cd_2_In_2_S_5_-based^65^ platforms. The low RSD (<1.4%) reflects superior precision, particularly in wastewater, where matrix effects often compromise sensor reliability. Unlike MIP-based sensors,^57,62,64^ which require time-consuming polymerization, or carbon-based sensors,^56,59,61^ which suffer from fouling or pH sensitivity, the InSe QD/Ta_2_O_5_-GCE offers a scalable, user-friendly alternative. The sensor's potential for environmental monitoring is further underscored by its ability to address emerging concerns about antibiotic resistance, as highlighted by the inclusion of macrolides and chloramphenicol in the EU Water Framework Directive watchlist. These attributes, combined with its compatibility with portable electrochemical platforms, position the InSe QD/Ta_2_O_5_-GCE as a promising candidate for routine monitoring and potential commercial applications. Detailed performance metrics and comparisons are provided in Table 5.

## 4. Conclusion

The InSe QD/Ta_2_O_5_-modified electrochemical sensor demonstrates superior performance for detecting antibiotic residues, including tetracycline, ciprofloxacin, amoxicillin, erythromycin, and chloramphenicol, in environmental matrices. With an ultralow limit of detection (2.55 × 10^−11^ M), the sensor outperforms other nanomaterial-based sensors, such as carbon nanotube-modified electrodes (LOD ∼1.8–5 nM) and graphene-based platforms (LOD ∼0.36–0.54 nM), due to the synergistic electrocatalytic activity of InSe quantum dots and the high surface area (520 m^2^ g^−1^) of mesoporous Ta_2_O_5_. Its exceptional stability (RSD <2% over 30 days) surpasses carbon nanotube sensors (∼3–5% RSD), attributed to the robust Ta_2_O_5_ matrix preventing QD aggregation. The sensor achieves high recovery rates (92.2–98.5%) in complex matrices like wastewater, compared to 85–95% for Fe-MOF or TiO_2_-based sensors, with minimal interference (<8.3%) from environmental components. Reproducibility (RSD <1.4% across 30 electrodes) exceeds that of MIP-based sensors (∼2.8–4.0% RSD). This platform's ultrasensitivity, stability, and reproducibility position it as a transformative tool for real-time environmental monitoring, offering significant advantages over existing electrochemical sensors for precise antibiotic detection.

## Conflicts of interest

There are no conflicts to declare.

## Data Availability

The datasets supporting this study are available from the corresponding author upon reasonable request.
